# Predicting protein targets for drug-like compounds using transcriptomics

**DOI:** 10.1371/journal.pcbi.1006651

**Published:** 2018-12-07

**Authors:** Nicolas A. Pabon, Yan Xia, Samuel K. Estabrooks, Zhaofeng Ye, Amanda K. Herbrand, Evelyn Süß, Ricardo M. Biondi, Victoria A. Assimon, Jason E. Gestwicki, Jeffrey L. Brodsky, Carlos J. Camacho, Ziv Bar-Joseph

**Affiliations:** 1 Department of Computational and Systems Biology, University of Pittsburgh, Pittsburgh, Pennsylvania, United States of America; 2 Machine Learning Department, School of Computer Science, Carnegie Mellon University, Pittsburgh, Pennsylvania, United States of America; 3 Department of Biological Sciences, University of Pittsburgh, Pittsburgh, Pennsylvania, United States of America; 4 School of Medicine, Tsinghua University, Beijing, China; 5 Department of Internal Medicine I, Universitätsklinikum Frankfurt, Frankfurt, Germany; 6 Department of Pharmaceutical Chemistry, University of California, San Francisco, San Francisco, California, United States of America; Icahn School of Medicine at Mount Sinai, UNITED STATES

## Abstract

An expanded chemical space is essential for improved identification of small molecules for emerging therapeutic targets. However, the identification of targets for novel compounds is biased towards the synthesis of known scaffolds that bind familiar protein families, limiting the exploration of chemical space. To change this paradigm, we validated a new pipeline that identifies small molecule-protein interactions and works even for compounds lacking similarity to known drugs. Based on differential mRNA profiles in multiple cell types exposed to drugs and in which gene knockdowns (KD) were conducted, we showed that drugs induce gene regulatory networks that correlate with those produced after silencing protein-coding genes. Next, we applied supervised machine learning to exploit drug-KD signature correlations and enriched our predictions using an orthogonal structure-based screen. As a proof-of-principle for this regimen, top-10/top-100 target prediction accuracies of 26% and 41%, respectively, were achieved on a validation of set 152 FDA-approved drugs and 3104 potential targets. We then predicted targets for 1680 compounds and validated chemical interactors with four targets that have proven difficult to chemically modulate, including non-covalent inhibitors of HRAS and KRAS. Importantly, drug-target interactions manifest as gene expression correlations between drug treatment and both target gene KD and KD of genes that act up- or down-stream of the target, even for relatively weak binders. These correlations provide new insights on the cellular response of disrupting protein interactions and highlight the complex genetic phenotypes of drug treatment. With further refinement, our pipeline may accelerate the identification and development of novel chemical classes by screening compound-target interactions.

## Author summary

Bioactive compounds often disrupt cellular gene expression in ways that are difficult to predict. While the correlation between a cellular response after treatment with a small molecule and the knockdown of its target protein should be simple to establish, in practice this goal has been difficult to achieve. The main challenges are that data are noisy, drugs are not intended to be active in all cell types, and signals from a bona fide target(s) may be obscured by correlations with knockdowns of other proteins in the same pathway(s). Here, we find that a random forest classification model can detect meaningful correlational patterns when gene expression profiles after compound treatment and gene knockdowns in four or more cell lines are compared. When this approach is enriched by a structure-based screen, novel drug-target interactions can be predicted. We then validated new ligand-protein interactions for four difficult targets. Although the initial compounds are not especially potent *in vitro*, they are capable of disrupting their target pathway in the cell to an extent that generates a significant and characteristic gene expression profile. Collectively, our studies provide insight on cell-level transcriptomic responses to pharmaceutical intervention and the use of these patterns for target identification. In addition, the method provides a novel drug discovery pipeline to test chemistries without a priori knowledge of their target(s).

## Introduction

Most research programs focus on a subset of roughly 10% of human proteins, and this bias has a profound effect on drug discovery, as exemplified by studies on protein kinases [1–3]. The origin for this relatively limited exploration of the human interactome and the resulting lack of novel drugs for emerging ‘genomic-era’ targets has been traced back to the availability of small molecular weight probes for only a narrow set of familiar protein families [1]. To break this vicious cycle, a new approach is needed that goes beyond known targets and old scaffolds and benefits from the vast amount of information we possess on gene expression, protein interactions, protein structures, and the genetic basis of disease.

The current target-centric paradigm relies on high-throughput *in vitro* screens of large compound libraries against a single protein [4]. This approach has been effective for kinases, GPCRs, and proteases, but has produced meager yields for new targets such as protein-protein interactions, which require chemotypes absent in most compound libraries [5, 6]. Moreover, these *in vitro* biochemical screens often cannot provide any context regarding drug activity in the cell, multi-target effects, or toxicity [7, 8]. On the other hand, the goal of leveraging new chemistries requires a compound-centric approach that would test compounds directly on thousands of potential targets. In practice, this is undertaken in cell-based phenotypic assays, but it is often unclear how to identify potential molecular targets in these experiments [9–11]. Understanding how cells respond when specific interactions are disrupted is not only essential for target identification but also for developing therapies that might restore perturbed disease networks to their native states.

Compound-centric computational approaches are now commonly applied to predict drug—target interactions by leveraging existing data. However, many of these methods extrapolate from known chemistry, structural homology, and/or functionally related compounds, and excel in target prediction only when the query compound is chemically or functionally similar to known drugs [12–17]. Other structure-based methods, such as molecular docking, can evaluate novel chemistries but are limited by the availability of protein structures [18–20], inadequate scoring functions, and excessive computing times, which render structure-based methods ill-suited for genome-wide virtual screens [21].

More recently, a new paradigm to predict molecular interactions using cellular gene expression profiles has emerged [22–24]. Previous work showed that distinct inhibitors of the same protein target produce similar transcriptional responses [25]. Other studies predicted secondary pathways affected by chemical inhibitors by identifying genes that, when deleted, diminish the transcriptomic signature of drug-treated cells [26]. When target information is lacking for a compound, alternate approaches were needed to map drug-induced differential gene expression networks onto known protein interaction network topologies. Prioritized potential targets could then be identified through highly perturbed subnetworks [27–29]. These studies predicted roughly 20% of known targets within the top 100 ranked genes, but did not predict or validate any previously unknown interactions.

**Fig 1:**
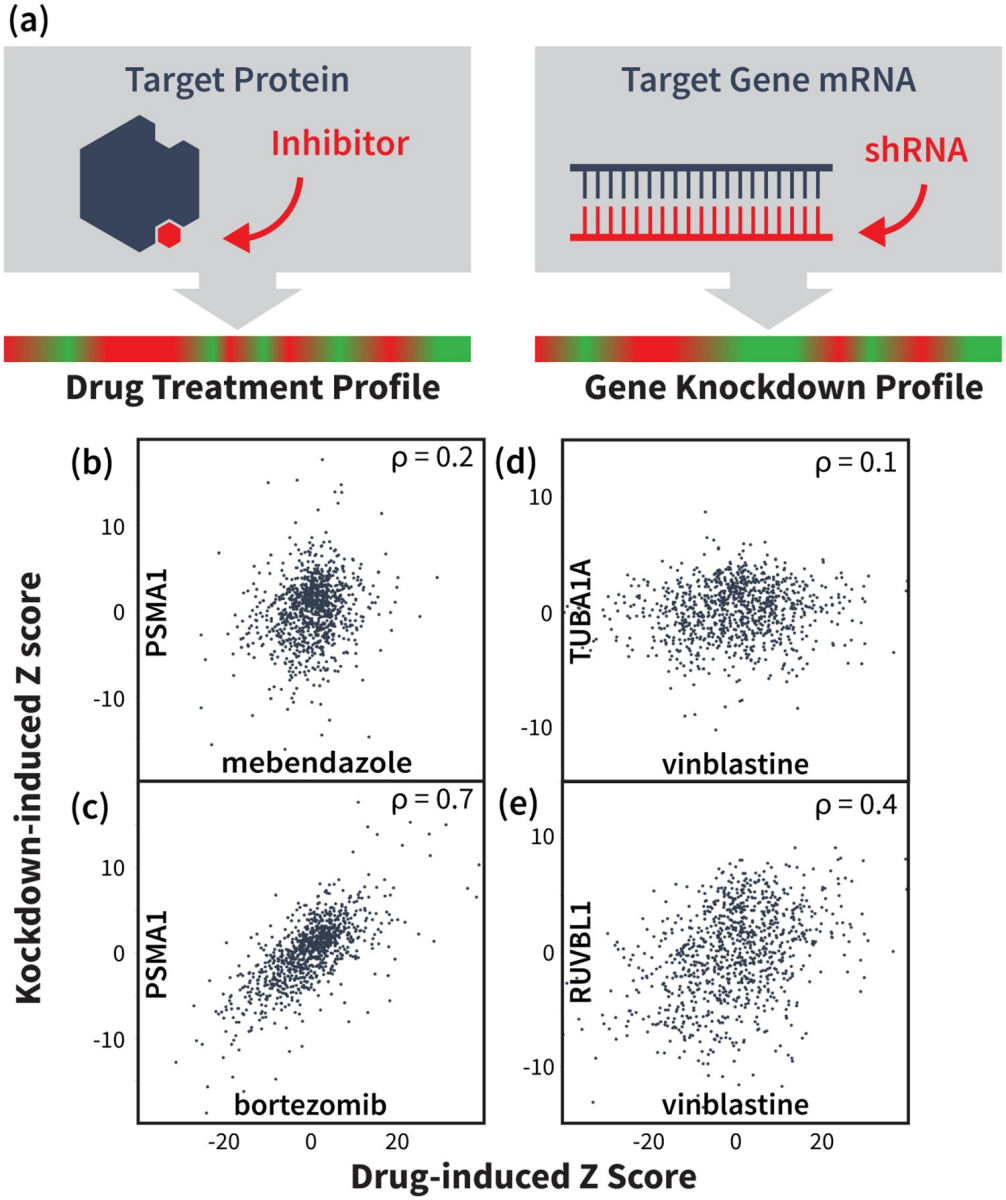
Drug and gene knockdown induced mRNA expression profile correlations reveal drug-target interactions. (a) Illustration of our main hypothesis: we expect a drug-induced mRNA signature to correlate with the knockdown (KD) signature of the drug’s target gene and/or genes on the same pathway(s). (b,c) mRNA signature from KD of proteasome gene PSMA1 does not significantly correlate with signature induced by tubulin-binding drug mebendazole, but shows strong correlation with signature from proteasome inhibitor bortezomib. Data points represent differential expression levels (Z-scores) for the 978 landmark genes measured in the LINCS L1000 experiments. (d,e) Signature from tubulin-binding drug vinblastine shows little signature correlation with KD of its target TUBA1A, but instead correlates with the KD of functionally related genes, such as RUVBL1.

The NIH Library of Integrated Cellular Signatures (LINCS) project presents an opportunity to leverage gene expression signatures from numerous cellular perturbations to predict drug-target interaction. Specifically, the LINCS L1000 dataset contains cellular mRNA signatures from treatments with over 20,000 small molecules and 20,000 gene over-expression (cDNA) or knockdown (sh-RNA) experiments. Based on the hypothesis that drugs which inhibit their target(s) should yield similar network-level effects to silencing the target gene(s) (Fig 1a), we calculated correlations between the expression signatures of thousands of small molecule treatments and gene knockdowns (KDs) in the same cells. We next used the strength of these correlations to rank potential targets for a validation set of 29 FDA-approved drugs tested in the seven most abundant LINCS cell lines. We then evaluated both direct signature correlations between drug treatments and KDs of their potential targets, as well as indirect signature correlations with KDs of proteins up- or down-stream of potential targets. We subsequently combined these correlation features with additional gene annotation, protein interaction and cell-specific features in a supervised learning framework and use Random Forest (RF) [30, 31] to predict each drug’s target. Ultimately, we achieved a top 100 target prediction accuracy of 55%, which we show is due primarily to our novel correlation features. Finally, to filter out false positives and further enrich our predictions, molecular docking evaluated the structural compatibility of the RF-predicted compound—target pairs. This orthogonal analysis significantly improved prediction accuracy on an expanded validation set of 152 FDA-approved drugs, obtaining top-10 and top-100 accuracies of 26% and 41%, respectively, more than double that of aforementioned methods. A receiving operating characteristic (ROC) analysis yielded an area under the curve (AUC) for top ranked targets of the RF and structural re-ranked predictions of 0.77 and 0.9, respectively. We then applied our pipeline to 1680 small molecules profiled in LINCS and experimentally validated seven potential first-in-class inhibitors for disease-relevant targets, namely HRAS, KRAS, CHIP, and PDK1.

## Results

### Preliminary prediction of drug targets using expression profile correlation features

We constructed a validation set of 29 FDA-approved drugs tested in at least seven LINCS cells lines and whose known targets were among 2634 KD genes in the same cell lines. For these drugs, we ranked potential targets using the direct correlation between the drug-induced mRNA expression signature and the KD-induced signatures of potential targets (Fig 1b and 1c). For each cell line, the 2634 KD signatures were sorted by their Pearson correlation with the expression signature of the drug in that cell line. We used each gene’s lowest rank across all cell lines to produce a final ranking of potential targets for the given drug. Using this approach, we predicted known targets in the top 100 potential targets for 8/29 validated compounds (Table 1). Indirect correlations were evaluated by the fraction of a potential target’s known interaction partners (cf. BioGrid [32]) whose KD signatures correlated strongly with the drug-induced signature. Ranking by indirect correlations predicted the known target in the top 100 for 10 of our 29 validation compounds (Table 1). Interestingly, several of these compounds showed little correlation with the KD of their targets (Fig 1d and 1e), with only 3/10 targets correctly predicted using the direct correlation feature alone.

**Table 1:** Performance of target prediction using different features and methods on the 29 FDA-approved drugs tested in 7 cell lines.

Drug	LINCS ID (BRD-)	Target(s)	Random	DIR	IND	CS	MAX	MEAN	LR	RF
**vinorelbine**	K10916986	TUBB6, TUBA1A, TUBB2A, TUBB2C	310	126	128	1318	1690	425	28	88
**dexamethasone**	A69951442	NR3C1	1498	1891	284	943	315	1143	757	157
**dasatinib**	K49328571	LCK, YES1	2325	1009	94	222	290	2621	182	532
**vincristine**	A76528577	TUBB6, TUBA1A, TUBB2A, TUBB2C	1979	473	439	386	2231	2196	456	37
**mycophenolate-mofetil**	K92428153	IMPDH2	564	1100	1263	2986	100	301	3064	3086
**amlodipine**	A22032524	CACNA1D	995	1338	2439	1801	1875	974	3037	650
**lovastatin**	A70155556	HMGCR	1712	72	811	2078	1124	1068	1334	55
**clobetasol**	A26095496	NR3C1	2194	820	21	157	74	15	38	65
**calcitriol**	K27316855	VDR	2514	1059	2938	221	125	1814	1299	252
**flutamide**	K28307902	AR	919	2604	69	2806	463	298	702	647
**prednisolone**	A27887842	NR3C1	2382	1439	206	787	402	1068	257	23
**nifedipine**	K96354014	CACNA1D	940	1225	1465	1285	88	322	3037	2249
**vemurafenib**	K56343971	BRAF	1042	1	82	1	1149	1403	22	2
**glibenclamide**	K36927236	KCNJ11	29	1415	2028	409	1059	740	1300	366
**digoxin**	A94756469	ATP1A3, FXYD2, ATP1B1	2376	73	1470	118	828	567	732	44
**bortezomib**	K88510285	PSMB10, PSMA3, PSMA1, PSMA5, PSMB7, PSMB5, PSMA8, PSMB1	1882	1	1	2	2546	2513	24	5
**vinblastine**	A22783572	TUBB6, TUBA1A, TUBB2A, TUBB2C	1612	515	56	100	224	377	38	2
**digitoxin**	A93236127	ATP1A3, FXYD2, ATP1B1	573	89	430	216	521	653	79	50
**losartan**	K76205745	AGTR1	645	489	988	770	636	31	735	1931
**pitavastatin**	K30097969	HMGCR	1855	1976	1036	1117	90	527	1632	373
**digoxin**	A75144621	ATP1A3, FXYD2, ATP1B1	69	521	776	194	127	559	208	64
**hydrocortisone**	A65767837	NR3C1	303	312	72	58	93	122	29	17
**paclitaxel**	A28746609	TUBB6, TUBA1A, TUBB2A, TUBB2C	2299	74	121	47	371	1862	79	19
**lovastatin**	K09416995	HMGCR	988	1	735	1587	1698	1484	128	100
**irinotecan**	K08547377	TOP1	1742	1023	20	236	128	1886	46	160
**vincristine**	A60414806	TUBB6, TUBA1A, TUBB2A, TUBB2C	1394	96	74	17	1272	69	28	9
**vinblastine**	A55594068	TUBB6, TUBA1A, TUBB2A, TUBB2C	1359	490	75	1383	373	1735	35	2
**raloxifene**	K63828191	ESR2	2080	2883	1818	1172	1064	479	1114	2520
**digoxin**	K23478508	ATP1A3, FXYD2, ATP1B1	1005	102	1066	112	2096	2027	252	167
		**Mean Ranking**	1365	800.6	724.3	776.9	794.9	1009.6	712.8	471.4
		**Top 100**	2	8	10	6	5	3	11	16

DIR: direct correlation feature; IND: indirect correlation feature; CS: cell selection feature; MAX: maximum differential expression feature; MEAN: mean differential expression feature; LR: logistic regression; RF: random forest. Values are for the ranking of the top known target for each drug.

It is well known that expression profiles vary between cell types [33]. Thus, we constructed a cell selection feature to determine the most “active” cell line, defined as the cell line producing the lowest correlation between the drug-induced signature and the control signature. Ranking by direct correlations within the most active cell line for each drug predicted six known targets in the top 100 (Table 1). However, all six of these targets were already predicted by either direct or indirect correlations, strongly suggesting that scanning for the optimal correlation across all cell lines is a better strategy than trying to identify the most relevant cell type by apparent activity.

Next, to incorporate findings of previous studies that suggest that drug treatments often up/down regulate the expression of their target’s interaction partners [27–29], we constructed two features to report directly on the drug-induced differential expression of potential target interaction partners. These features compute the maximum and the mean differential expression levels of potential interaction partners in the drug-induced expression profile. The lowest rank of each potential target across all cell lines is used in a final ranking. Though neither expression feature produces top 100 accuracies better than those of our correlation features, maximum differential expression identifies three new targets that were not identified using any of the previous features (Table 1).

### Combining individual features using random forest

While each of the features in Table 1 performed better than random, combining them further improved results. Using Leave-One-Out Cross Validation (LOOCV) for each drug, logistic regression [31] correctly identified known targets in the top 100 predictions for 11 out of 29 drugs and improved the average known target ranking of all drugs (Table 1). However, logistic regression assumes that features are independent, which is not the case for our dataset given the complexity and density of cellular protein interaction networks. Hence, we used RF, which is able to learn more sophisticated decision boundaries [34]. Following the same LOOCV procedure, the RF classifier led to much better results than the baseline logistic regression, correctly finding the target in the top 100 for 16 out of 29 drugs (55%) (Table 1). Without further training, we tested the RF approach on the remaining 123 FDA-approved drugs that had been profiled in 4, 5, and 6 different LINCS cell lines, and whose known targets were among 3104 genes knocked down in the same cells. We predicted known targets for 32 drugs (26%) in the top 100 (S2 Text), an encouraging result given the relatively small size of the training set and the expected decline in accuracy as the number of cell lines decreases (Table 2).

**Table 2:** Performance of two random forest models on validation set of 152 FDA-approved drugs as a function of cells tested.

**# of Cells**	**All**	**7**	**6**	**5**	**4**
**# of Drugs**	152	29	30	42	51
**On-the-fly**					
Top 100	58	13	15	16	14
Top 50	42	10	10	12	10
Top 100%	38%	45%	50%	38%	27%
Top 50%	28%	34%	33%	29%	20%
**Two-level**					
Top 100	63	14	15	22	13
Top 50	54	12	14	20	8
Top 100%	41%	48%	50%	52%	25%
Top 50%	36%	41%	47%	48%	16%

Top 50%/100% - percent of drugs with targets correctly predicted as top 50/100. The number of drugs with targets ranked in top 100/50 are shown for the “on-the-fly” and “two-level” RF classification models. Results of the models are shown for “All” drugs tested in four or more cell lines, as well as for the subsets of drugs profiled in different numbers of cell lines. Note that the success rate for RF is significant with p < 10^−6^ based on randomization tests (S1 Fig).

Re-training on the full set of 152 drugs and validating with LOOCV allowed us to test two alternative RF models: “on-the-fly”, which learns drug-specific classifiers trained on the set of drugs profiled in the same cell types, and “two-level”, which learns a single classifier trained on experiments from all training drugs (see Methods). The performances of both methods as a function of the number of cell lines profiled are summarized in Table 2. On-the-fly RF correctly ranked the targets of 8 out of 152 drugs in the top 100 (38%), with 42 of them in top 50 (28%). Two-level RF produced better enrichment, correctly predicting targets for 63 drugs in the top 100 (41%), and for 54 drugs in the top 50 (36%).

To further evaluate model performance, we generated a receiver operating characteristic (ROC) curve from the LOOCV predictions of our two-level RF (S2 Fig). In this analysis, the False Positive Rate (x axis) is the normalized rank threshold we use to define potential targets from non-targets (e.g., top-10, top-100). The True Positive Rate (y axis) is the fraction of compounds for which the known target ranks above the given threshold. Prediction power is measured as the area under the ROC curve (AUC), with AUC = 1 indicating perfect prediction and AUC = 0.5 indicating random prediction. Our RF produced an AUC of 0.77 while, in sharp contrast, random rankings (based on 20000 permutations) leads to only 7% of drugs with targets in the 100, indicating that both our training/testing and LOOCV results are extremely significant (S1 Fig). It is also noteworthy that the top-100 accuracy of the two-level RF analysis increases to 50% if we only consider drugs treated in 5 or more cell lines.

We note that 20%, or 33, out of the 152 FDA approved training drugs have multiple known targets with KD signatures in the LINCS library (S1 Table). However, only 16 of those had more than one target among the KDs in four or more cell lines. Thus, only a small portion (10%) of the analyzed compounds had multiple known targets that we could potentially predict (see S2 Table), making the analysis of polypharmacological effects difficult. However, for 4 (out of 16) multi-target compounds, our RF model was able to identify more than one target. It is thus possible that drugs in the training set might bind to other targets that could be in our top 100 list.

### Gene ontology analysis of protein targets

Next, we analyzed in what context our RF analysis was most successful. To this end, we divided the 152 drugs in our training data into “successful” predictions (the 63 drugs for which the correct target was ranked in the top 100), and “unsuccessful” predictions. We also divided the known targets into those that were correctly predicted and those that were not. We considered several different ways to characterize small molecules including molecular weight, solubility, and hydrophobicity, but none of these seemed to significantly correlate with our “successful” and “unsuccessful” classifications. Next, we used gene ontology to test for enrichment of “successful” and “unsuccessful” targets. Interestingly, we found that “successful” targets were significantly associated with intracellular categories, while the “unsuccessful” targets were mostly associated with transmembrane and extracellular categories (S3 Table). Based on this result we further incorporated this cellular component as a feature in our two-level RF. We encode this feature by assigning 1 to the intracellular genes and -1 to the transmembrane and extracellular ones. We ran the two-level RF with this additional feature included and demonstrated that the cellular component increases the number of top 100 genes to 66 and top 50 genes to 55. These results demonstrate the possibility of further improving our predictions by incorporating relevant properties of compounds or targets.

### Structural enrichment of genomic predictions

Fig 1d and 1e show that the gene regulatory effects of TUBA1A inhibition by the drug vinblastine manifest primarily as indirect correlations with KDs of the target’s interaction partners, such as RUVBL1, rather than via direct correlation with KD of the target. Such cases reflect the intrinsic connectivity of cellular signaling networks, which sometimes produce gene expression correlations that are ambiguous with respect to which of the interacting proteins in the affected pathway is the drug’s actual target. Our pipeline eliminates some of these false positives using an orthogonal structure-based docking scheme that—although limited to targets with known structure—allows us to significantly improve our prediction accuracy. After performing RF classification on the validation set, we mined the Protein Data Bank (PDB) [35] to generated structural models of the potential targets for our 63 “hits”. This set represents drugs for which we correctly identified the known target in the top 100. We selected one or more representative crystal structures for each potential target gene, optimizing for sequence coverage and structural resolution (see S1 Text). We then docked hits to their top 100 potential targets and ranked them using a prospectively validated pipeline [36–39].

**Fig 2:**
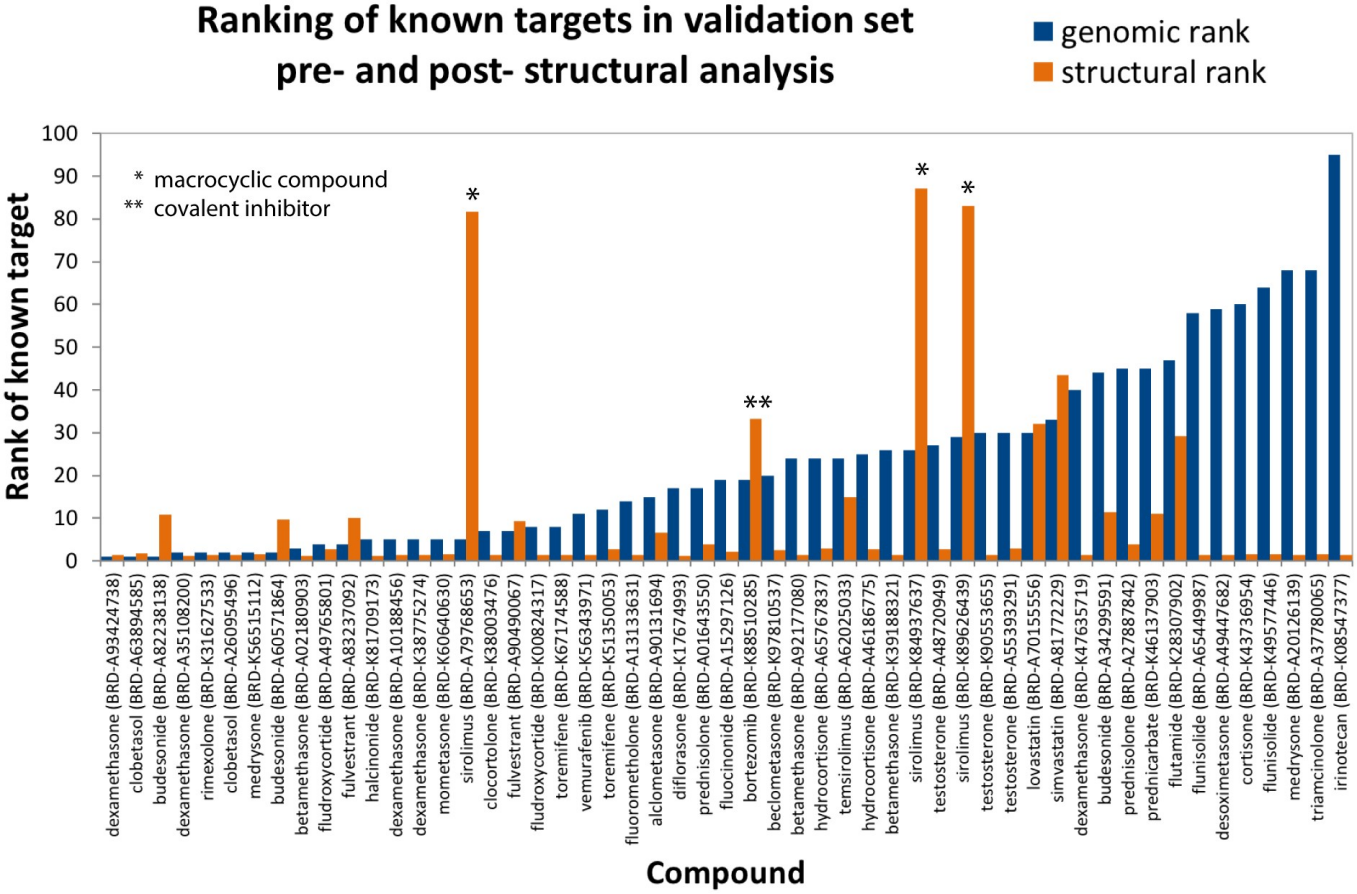
Structural enrichment of genomic target predictions. Predicted ranking (lower is better) of the highest-ranking known target for the 53 hits in our validation set with known target structures. Percentile rankings are shown following RF analysis (blue) and following structural re-ranking (orange). We note that docking and scoring macrocycles or covalent inhibitors is particularly challenging. Furthermore, scoring functions are destined to predict false positives, yet within the limited and orthogonal set of drug targets predicted by the genomic screening the scoring function used in our pipeline [36] shows significant enrichment.

On average, crystal structures were available for 69 out of the top 100 potential targets for each compound, and structures of known targets were available for 53 of the 63 hits. In order to avoid redocking into cocrystals, we excluded all crystal structures containing these 53 ligands from our analysis, ensuring that our results would not depend on prior knowledge of interaction partners or binding modes. As shown in Fig 2, molecular docking scores improved re-ranking of the known target for 40 of the 53 drugs, with a mean and median improvement of 13 and 9, respectively. Based on genomic data alone, the known target was ranked in the top 10 for 40% of the 63 hits. After structural re-ranking, 65% had their known targets in the top 10 candidates, and this value improved to 75% in the subset of 53 drugs with known target structures. ROC analysis of structurally-refined predictions yielded an AUC of 0.90 (S3 Fig). These results demonstrate the power of orthogonal genomic and structural screens and establish that molecular docking can efficiently eliminate false positives in our gene expression-based predictions.

### Identifying new interactions in the LINCS dataset

**Fig 3:**
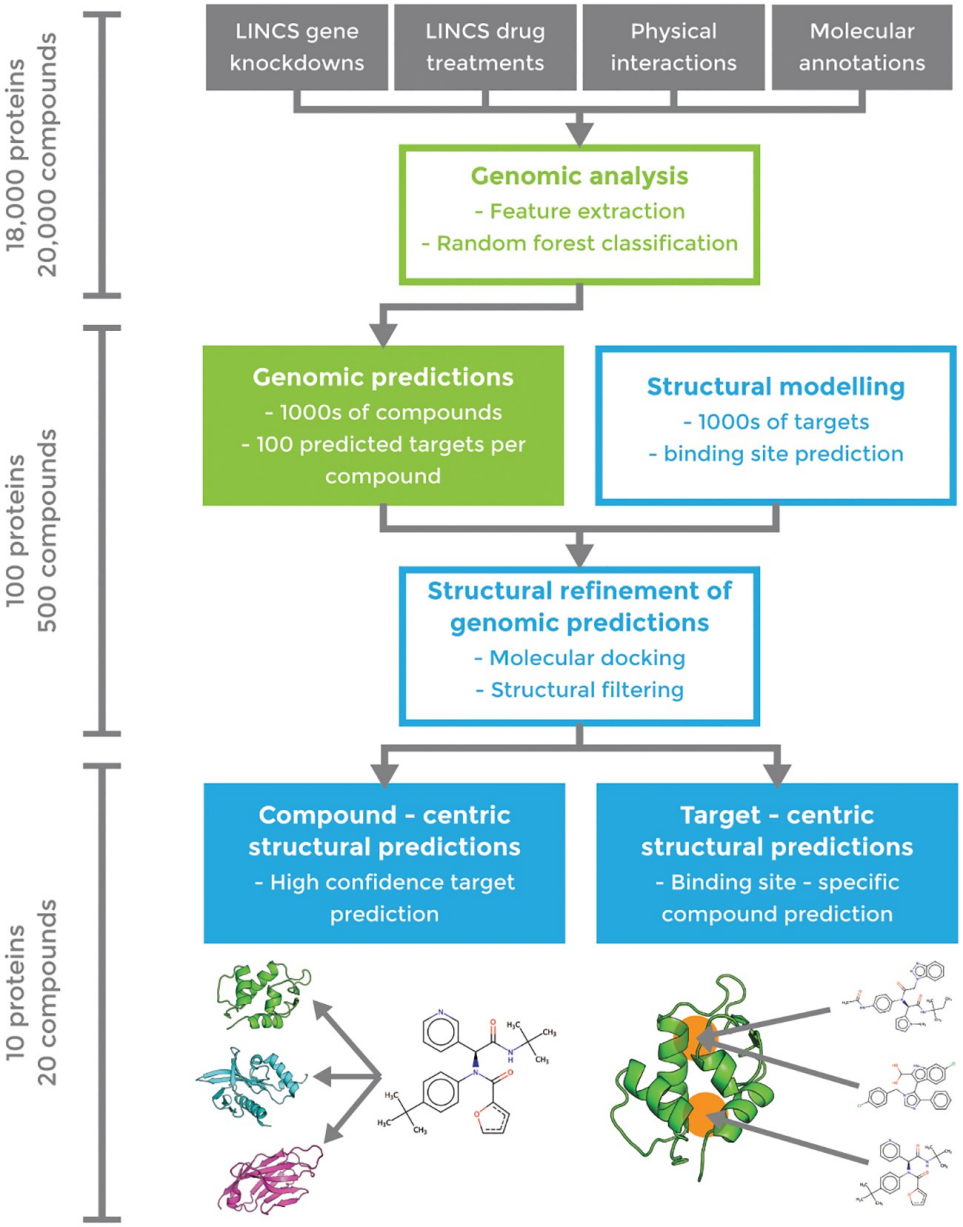
Workflow of combined genomic (green) and structural (blue) pipeline for drug-target interaction prediction. Approximate numbers of proteins/compounds in each phase are indicated on the left.

After validating our approach on known drug targets, we applied our pipeline to a test set of 1680 small molecules and 3333 gene KDs and predicted several novel interactions. The experimental testing set was chosen based solely on the predicted correlations of the RF model and availability of the assays. We applied our pipeline (Fig 3) in both compound-centric (target prediction) and target-centric (virtual screening) contexts, in each case producing a final, enriched subset of roughly 10 predictions (either compounds or targets) that we tested experimentally. In compound-centric analyses, we performed molecular docking on the available structures of the input compound’s top-100 RF-predicted targets. In target-centric analyses, we ran the RF on our full test set, identified compounds for which the input protein is ranked in the top 100 potential targets, and then docked these candidates into the target. In both applications, we analyzed the final docking score distributions and applied a 50% cutoff threshold to identify highly enriched compound/target hits. Structural analysis further facilitated visual validation of the docking models of predicted hits, thereby minimizing false positives. Because of limitations in available assays for subsequent tests, we analyzed our experimental results within a target-centric approach. According to our validation results, we would expect one hit in about 5 to 6 compounds on targets where crystal structures are available. As outlined below, we chose four targets for this analysis, and it is vital to note that the compounds have not been optimized but represent “crude” hits obtained from the pipeline. Needless to say, significantly improved results could be obtained with chemical optimization, but our efforts simply represent a facile way to isolate these initial hits.

### Target-centric prediction of novel RAS inhibitors

Our first application consisted in identifying novel binders of the high-impact and historically “undruggable” RAS-family of oncoproteins. HRAS and KRAS are among the most frequently mutated genes in human cancers [40, 41]. However, despite the extensive structural data available and tremendous efforts to target them with small-molecule therapeutics, as of yet no RAS-targeting drug candidates have shown success in clinical trials [42–44].

**Fig 4:**
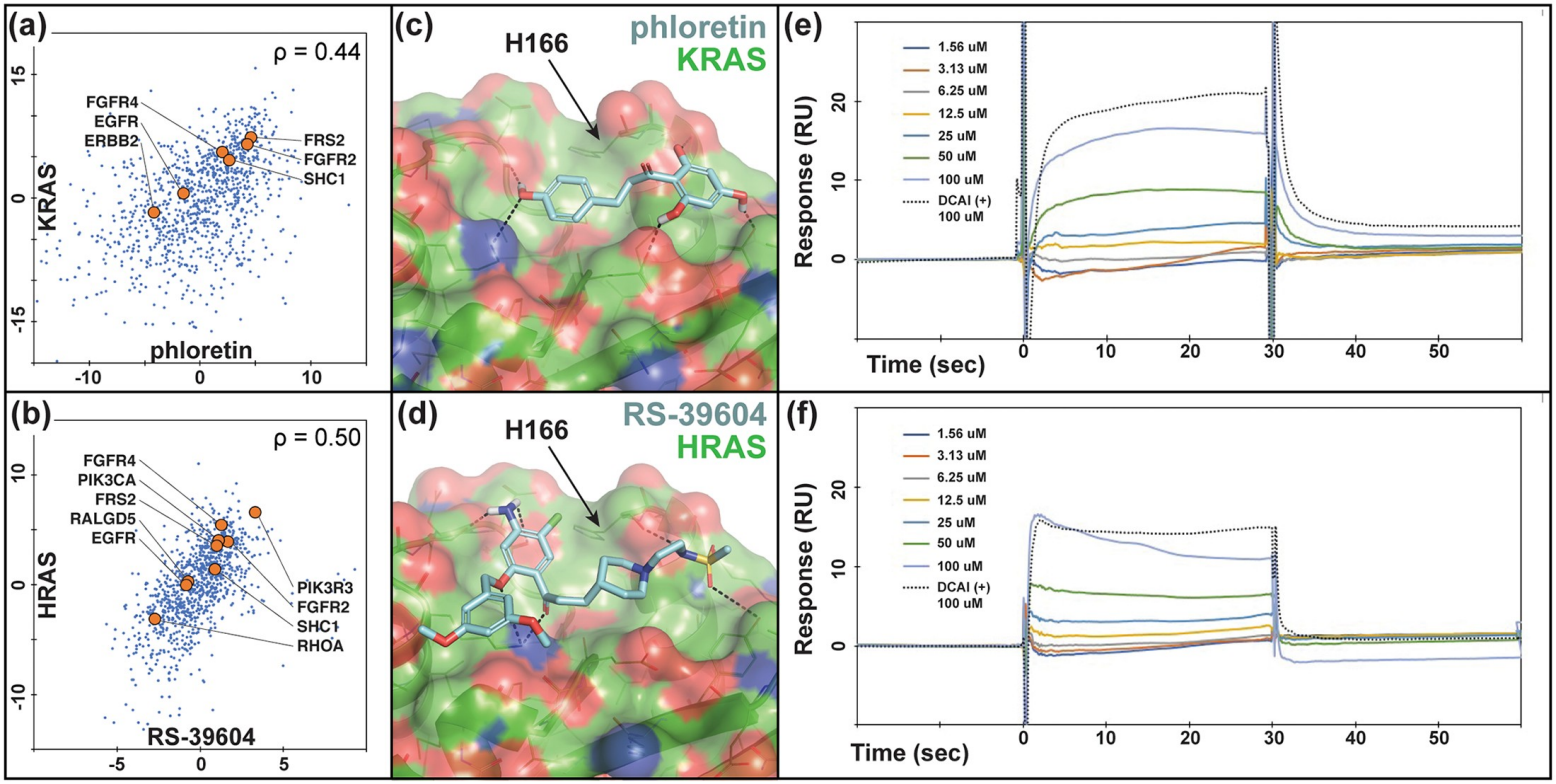
HRAS/KRAS inhibitors predicted based on direct correlations and docked poses show direct binding in SPR assays. Differential gene expression profiles of (a) Phloretin and (b) RS-39604 cell treatments and KRAS and HRAS KD experiments, respectively. Several functionally related genes listed in BioGrid [32] are indicated to demonstrate the relevance of these profiles as suggestive of direct drug-target interactions. Models of (c) phloretin and (d) RS-39604 bound to an allosteric site on the KRAS and HRAS catalytic domains, respectively. (e) SPR titration response curves for (e) phloretin and (f) RS-39604 binding to KRAS and HRAS, respectively, compared to DCAI positive control.

Among the 1680 compounds in our test set, 84 and 156 were predicted (within the top-100) to target KRAS and HRAS, respectively. These compounds produced mRNA perturbation signatures that correlated strongly with KDs of KRAS (Fig 4a), and HRAS (Fig 4b). Of note, differential expression of genes functionally related to K/HRAS, i.e. FGFR4, FGFR2, FRS1, inform on novel regulatory phenotypes responding to both compound inhibition and gene knock out. We docked predicted compounds to our representative structures of KRAS (PDB ID: 4DSO [42]) and HRAS (PDB ID: 4G0N [45]) (Fig 4c and 4d). RF ranking and docking score distributions were compared to select compounds from our enriched datasets that were both commercially available and moderately priced. Docking models of promising candidates were also examined visually to reject models with unmatched hydrogen bonds [46] and select those that showed suitable mechanisms of action (see, e.g., Fig 4c and 4d). We purchased six potential HRAS inhibitors and five potential KRAS inhibitors for experimental validation (S4 Table).

We sent our compounds to the RAS Biochemistry and Biophysics Group at Leidos Biomedical Research for validation. Their SPR assay measured direct binding of predicted inhibitors to AviTagged HRAS and KRAS. Initial 100 μM screens showed binding response for compounds RS-3906 against HRAS and phloretin against KRAS, and subsequent titrations confirmed binding at μM concentrations (Fig 4e and 4f), comparable to the DCAI positive control [42].

### Target-centric prediction of novel CHIP inhibitors

Next, we targeted STUB1, also known as CHIP (the carboxy-terminus of Hsc70 interacting protein), an E3 ubiquitin ligase that manages the turnover of over 60 cellular substrates [47]. To our knowledge, inhibitors of this ligase—even of low affinity/potency—have not been identified. CHIP interacts with the Hsp70 and Hsp90 molecular chaperones via its TPR motif, which recruits protein substrates and catalyzes their ubiquitination. Thus, treatment with small molecules that inhibit CHIP may prove valuable for pathologies where substrates are prematurely destroyed by the ubiquitin-proteasome system [48].

The screening of the 1680 LINCS small molecules profiled in at least four cell lines predicted 104 compounds with CHIP among the top 100 targets. We docked these molecules to our representative structure of the TPR domain of CHIP (PDB ID: 2C2L [49]), for which we had an available fluorescence polarization (FP) assay. The RF ranking and docking score distributions were compared to select compounds highly enriched in one or both scoring metrics. We next visually examined the docking models of top ranking/scoring hits to select those that show suitable mechanisms of action, and purchased six compounds for testing (S5 Table). In parallel, we performed a pharmacophore-based virtual screen of the ZINC database [50] using the *ZincPharmer* [39] server, followed by the same structural optimization [36–39] performed on the LINCS compounds. We purchased seven of the resulting ZINC compounds for parallel testing (none of the selected compounds were in the LINCS library).

**Fig 5:**
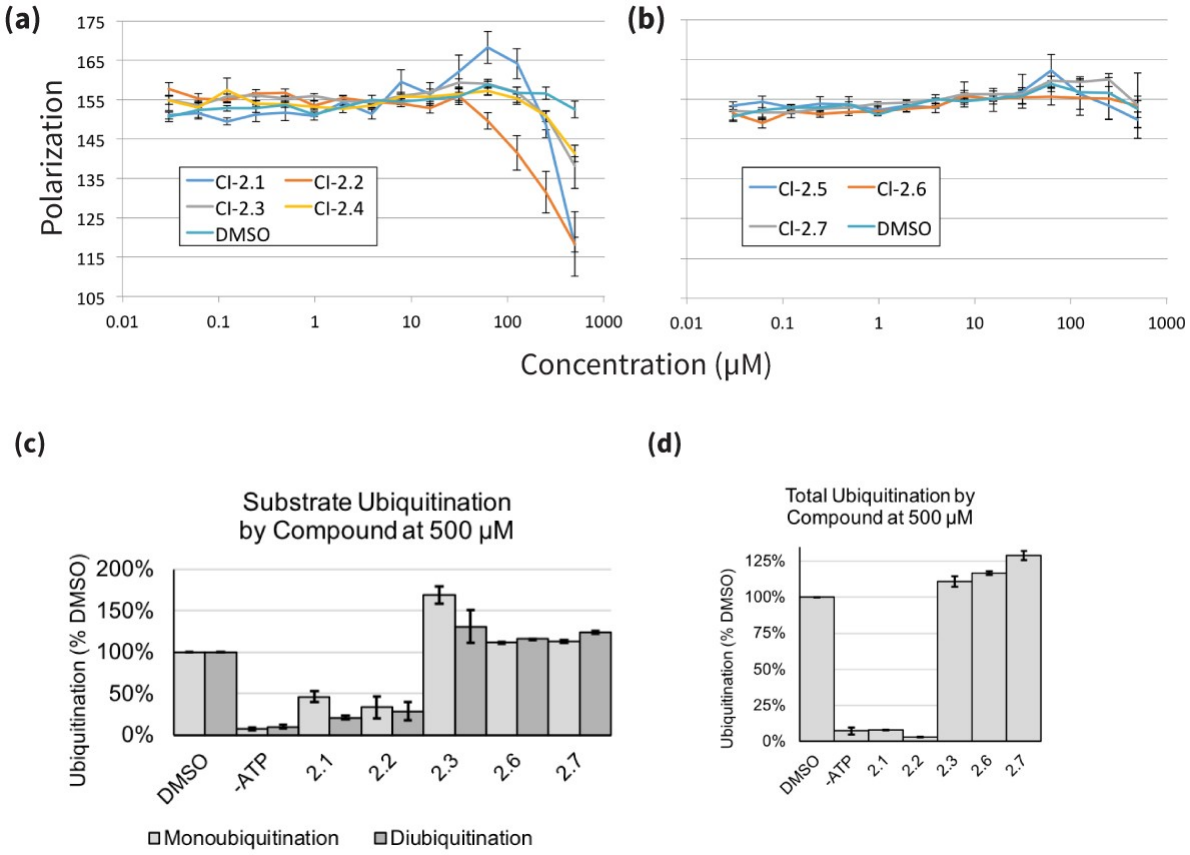
Predicted inhibitors show direct binding to and functional inhibition of CHIP. (a,b) Predicted CHIP inhibitors disrupt binding to chaperone peptide by fluorescence polarization. High ranked (a) and low ranked (b) compounds were tested for the ability to compete with a known TPR ligand (5-FAM-GSGPTIEEVD, 0.1 μM) for binding to CHIP (0.5 μM). Results are the average and standard error of the mean of two experiments each performed in triplicate. (c,d) CHIP inhibitors prevent ubiquitination by CHIP in vitro. (c) Quantification of substrate ubiquitination by CHIP from Anti-GST western blot experiments with tested compounds at 500 μM, blotted as in S5a Fig and normalized to DMSO treated control (2.1, 2.2: N = 4; all other compounds: N = 2). (d) Quantification of total ubiquitination by CHIP from Anti-GST western blot experiments with tested compounds at 500 μM, blotted as in S5b Fig and normalized to ubiquitination by a DMSO treated control (all compounds: N = 2).

Our FP assay measured competition with a natural peptide substrate for the CHIP TPR domain. We found that four (out of six) of our LINCS compounds reliably reduced substrate binding (Fig 5a and 5b), while three (out of seven) ZINC compounds did so to a modest degree (S4 Fig). The two strongest binders were LINCS compounds 2.1 and 2.2. To test if these compounds would inhibit CHIP activity, we utilized a cell-free ubiquitination assay in which purified CHIP polyubiquitinates an Hsc70-derived substrate protein in an ATP dependent manner (S5a Fig). This functional assay verified that 2.1 and 2.2 prevented substrate ubiquitination and CHIP autoubiquitination (Fig 5c and 5d, S5b and S5c Fig), while ZINC compounds did not (S5d Fig). Compounds 2.1 and 2.2 also prevented ubiquitination of an alternate substrate that was tested subsequently (S6 Fig). Importantly, the predicted binding modes of these two compounds did not match the pharmacophore model of the TPR-HSP90 interaction [49], which was used to screen the ZINC database (S7 Fig). The latter emphasizes the power of our approach to identify novel compounds and mechanisms of action to targets without known inhibitors.

**Fig 6:**
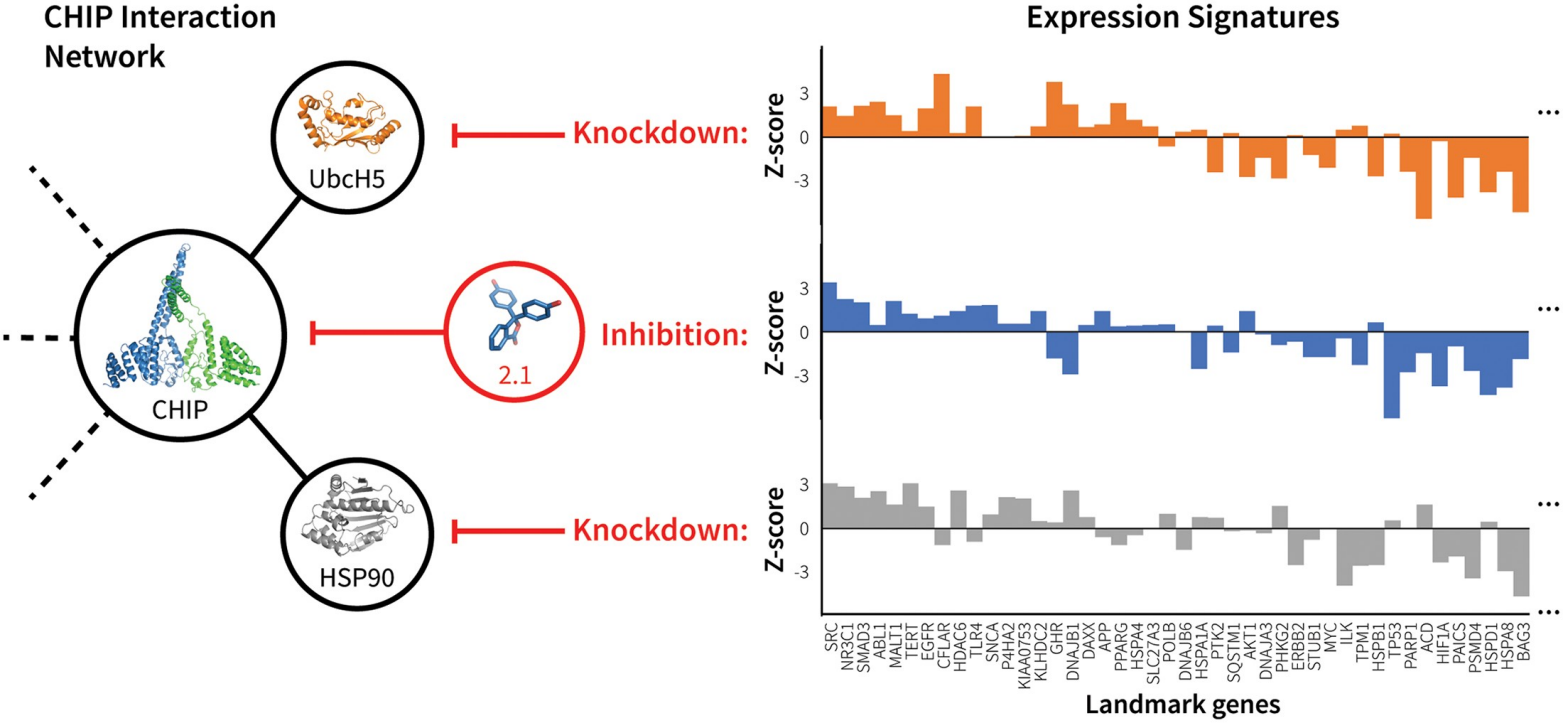
mRNA expression signature of CHIP inhibitor 2.1 correlates with knockdown of CHIP interacting partners. The figure illustrates the correlation between the mRNA expression profile signatures produced by treating cells with 2.1 and by knocking down CHIP interaction partners UbcH5 and HSP90. These three perturbations have similar network effects (left), as illustrated by their resulting differential expression signatures (right). For clarity, expression signatures show only the subset of LINCS landmark genes that are functionally related to CHIP according to BioGRID [32].

Contrary to the RAS compounds that were identified based on direct correlations between compound treatments and RAS KDs (Fig 4a and 4b), CHIP hits show almost no direct correlation (*ρ*_2.1_ = 0.15, *ρ*_2.2_ = 0.02), but were predicted based on indirect correlations with CHIP interaction partners. This may explain their relatively low potency. Fig 6 shows the correlating differential gene expression profiles for compound 2.1 and KDs of the CHIP interaction partners UbcH5 and HSP90, which, along with CHIP, were also predicted as potential targets by the RF classifier. However, structural screening ruled out these two partners as potential targets because of a lack of favorable binding modes.

### Compound-centric prediction of a novel target for the drug Wortmannin

**Fig 7:**
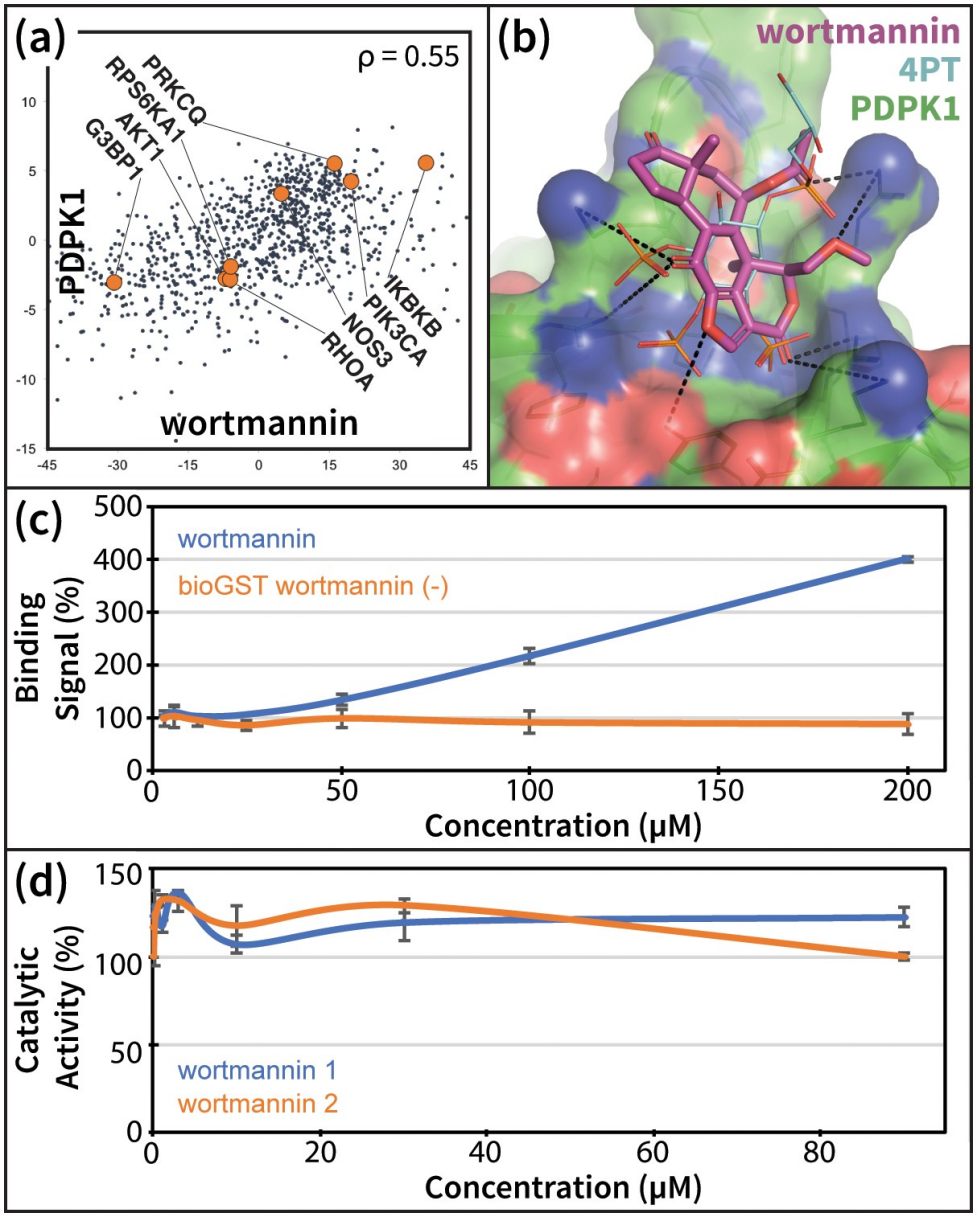
Wortmannin promotes PDK1 –PIP3 binding in vitro. (a) Wortmannin treatment and PDK1 KD experiments produce directly correlating differential gene expression profiles. Several functionally related genes listed in BioGrid [32] are indicated to demonstrate the relevance of these profiles as suggestive of direct drug-target interactions. (b) Model of wortmannin bound to the PH domain of PDK1, compared to known ligand 4PT (PDB ID: 1W1G [53]). (c) Alphascreen PDK1-PIP3 interaction-displacement assay results for increasing concentrations of wortmannin. Error bars represent the standard error of the mean from two parallel runs. (d) Effect of wortmannin on the in-vitro phosphorylation of the substrate T308tide [54] by the isolated catalytic domain of PDK1. The two lines are from two replicates of the activity assay, with error bars representing the standard error of the mean from two parallel runs for each replicate.

We next demonstrated a compound-centric application of our pipeline by analyzing Wortmannin, a selective PI3K covalent inhibitor and commonly used cell biological tool. DrugBank [51] lists four known human targets of Wortmannin: PIK3CG, PLK1, PIK3R1, and PIK3CA. Of the 100 targets predicted for Wortmannin, the PDB contained structures for 75, which we used to re-rank these potential targets. Only one known kinase target of Wortmannin, PIK3CA, was detected, and ranked 5^th^. The human kinase PDPK1 (PDK1) ranked 2^nd^ in our pipeline. Although PDK1 is a downstream signaling partner of the PI3Ks [52], there is no prior evidence of a direct Wortmannin-PDK1 interaction in the literature. Nevertheless, both the strong direct correlation of wortmannin with the PDK1 KD (Fig 7a), and the native-like binding mode predicted by our pipeline (Fig 7b) suggested a possible interaction.

We experimentally tested this interaction using an alphascreen PDK1 interaction-displacement assay. Since we predicted that Wortmannin binds to the PH domain of PDK1 (Fig 7b), we measured the effect of increasing Wortmannin concentrations on the interaction of PDK1 with the second messenger PIP3. We found that Wortmannin specifically increased PDK1-PIP3 interaction, relative to control (Fig 7c). Given that PIP3-mediated recruitment of PDK1 to the membrane is thought to play an important regulatory role in the activity of the enzyme [55, 56], a disruptive increase in PDK1-PIP3 interaction following treatment with Wortmannin supports our prediction.

### Comparison to existing target prediction methods

For completeness, we compared results for our 63 hits from the validation set to those produced by available structure and ligand-based methods. HTDocking (HTD) [57] is a structure-based target prediction method that docks and scores the input compound against a manually curated set of 607 human protein structures. For comparison, in our analysis we were able to extract high-quality domain structures for 1245 (40%) of the 3104 potential gene targets. PharmMapper (PHM) [58] is a ligand-based approach that screens the input compound against pharmacophore models generated from publicly available bound drug-target cocrystal structures of 459 human proteins, and then ranks potential targets by the degree to which the input compound matches the binding mode of the cocrystalized ligands. The scope of HTD is limited by the availability of the target structure, while PHM is limited by chemical and structural similarity of active ligands.

HTD and PHM rankings for known targets are shown in Table 3, and complete results are shown in S3 Text. Our combined genomics-structure method outperforms the structure-based HTD server (average ranking of the known target is 13 for our method vs. 50 for the HTD server). This observation suggests that limiting the structural screening to our genomic hits allowed us to predict targets with higher accuracy than docking alone. Results when using the PHM server are on average similar to ours. However, PHM relies on the availability of ligand-bound crystal structures, which in practice makes this class of methods more suitable for drug repurposing than assessing new chemistries or targets.

**Table 3:** Comparison of our pipeline to existing drug-target prediction methods.

	Structures available	Genomic Rank	Structural Re-rank	HTD	PHM
**All hits (n = 63)**	69	22	24	56	23
**Hits w/ known target structures (n = 53)**	71	23	13	50	12
**New predictions (n = 7)**	73	28	31	n/a	n/a

The average ranking of the highest ranked known target is listed for all 63 validation ‘hits’, for the subset of 53 validation hits with known target structures, and for our seven predicted interactions. ‘Structures available’ indicates the average number of top-100 potential targets with available crystal structures for the compound set. Rankings are compared between the initial random-forest genomic ranking, the structural re-ranking of the top 100 RF predicted targets, the HTDocking server (HTD), and the PharmMapper server (PHM).

Finally, we emphasize that alternative approaches failed to predict compound interactions with HRAS, KRAS, and CHIP that were verified—albeit with low potency—in our assays. However, a Wortmannin-PDK1 interaction was predicted at the catalytic site by HTD, ranked 540^th^, and by PHM, ranked 56^th^. Although we cannot rule out a possible kinase domain interaction, a catalytic activity assay showed that Wortmannin had no measureable effect on the in vitro phosphorylation of the substrate T308tide [54] by the isolated catalytic domain of PDK1 (Fig 7d). Overall, the novel drug-protein interaction pipeline outlined in this study can now be significantly improved—with ever-expanding genomic and proteomic databases—to continue to identify new probe compounds for specific protein targets. Even without further optimization, some of these probes can be used to test new hypotheses, as described above. Through medicinal chemistry, other probes can be in turn be optimized to provide more potent effects in cell and *in vitro*-based systems.

## Discussion

Delineating the role of small molecules in perturbing cellular interaction networks in normal and disease states is an important step towards identifying new therapeutic targets and chemistries for drug development. To advance toward this goal, we developed a novel target prediction method based on the hypothesis that drugs that inhibit a given protein should have similar network-level effects to silencing the inhibited gene and/or its up- or down-stream partners. Using gene expression profiles from KD and drug treatment experiments in multiple cell types from the LINCS L1000 dataset, we developed several correlation-based features and combined them in a RF model to predict drug-target interactions. Notably, the identified candidates validated our hypothesis that drug treatments and target KDs cause similar disruptions of cellular protein networks. More interestingly—and consistent with our hypothesis—we discovered that these correlations occur for KDs of the drug’s actual protein target(s) and/or for genes up- or down-stream of the target(s). We refer to the latter as “indirect correlations”. Several aspects of our approach represent a significant step forward from previous work exploring only expression correlations as a means to predict molecular interactions [59, 60]. In our case, there are no assumptions about the small molecule or its likely protein target/pathway, and our evaluation of both direct and indirect correlations allow us to screen compounds on a much larger scale and with higher accuracy than previously reported. Furthermore, to our knowledge, this is the first time that pathway connectivity is explicitly considered by indirect correlational effects between drugs and KDs of target interaction partners. Importantly, we have also open-sourced our predictions and methods, providing enriched sets of what will undoubtedly lead to active compounds for hundreds of human targets. In more general terms, our approach presents a new avenue for identifying suitable targets for novel chemistries, accelerating the discovery of chemical probes and potentially new drugs.

On a validation set of 152 FDA-approved drugs, we achieve top-100 target prediction accuracy more than double that of previous approaches that use differential expression alone [28, 29]. Consistent with our underlying hypothesis, the RF results highlight the importance of both direct expression signature correlations between drug treatment and KD of the gene target (Figs 1c, 4a, 4b and 7a) and indirect correlations between the drug and the target’s interacting partners (Figs 1e and 6). Contrary to earlier work [27–29], our method is capable of predicting potential targets for any compound, even those unrelated to known drugs, and as noted above, our predictions are open source and available for immediate download and testing (http://sb.cs.cmu.edu/Target2/). These include potential targets for 1680 LINCS small molecules from among over 3000 different human proteins.

Unlike most available ligand-based prediction methods [12–17], the accuracy of our approach does not rely on chemical similarity between compounds in the training/test sets. For instance, our screen against CHIP, a target with no known small molecule inhibitors, delivered four out of six binding compounds, whereas a parallel analysis using a state-of-the-art structure-based virtual screening [36, 61] yielded even weaker-binding compounds. Moreover, the predicted mechanisms of actions of the more potent LINCS compounds suggest novel interactions that were not prioritized by the ligand-based screen (S7 Fig).

In contrast to other machine learning methods, our approach reveals important, human-interpretable insights into perturbation-response properties of cellular networks. Direct and indirect gene expression profile correlations inform on global regulatory responses triggered by small molecule cell treatments (see, e.g., Figs 4, 6 and 7). Namely, our genomic screening not only identifies compounds targeting a given protein, but also highlight related genes that are affected by the chemical modulation of the target. This knowledge is bound to play an important role in the design of polypharmacological therapies.

Detailed analyses of our predictions suggest several avenues to improve enrichment. First, we established a clear correlation between the number of cell-types screened and the target prediction accuracy. Second, we identified that a significant source of false positives are indirect correlations that, while important to detect the true target, also tend to predict interacting partners as potential targets. Incorporating compound- or target-specific features are also likely to improve our results. For instance, we noticed that our prediction results were less accurate for extracellular and membrane proteins, and incorporating a cellular localization feature into our RF model increased the number of top-100 hits in our validation set from 63 to 66. Third, we envision that improved KD databases and transcriptomic profiling databases will emerge, as will more entries and higher resolution structures into the PDB, leading to more effective computational strategies. Nevertheless, we are aware that our pipeline currently suffers from several limitations. For example, since the LINCS data is currently based on 978 landmark genes, any correlations that are not reflected by these genes (which may be identified when using the full list of 20K genes) will be lost. Moreover, LINCS has only profiled genes in a small number of cell lines. While we try to account for this limitation with special features, some targets are likely missed because of inactivity in these cell lines. As noted above, we expect to improve on many of these issues when new LINCS data are released as this should include more KDs in more cell lines. A more detailed analyses of polypharmacological effects could also improve predictions, and we are aware that this will likely occur, especially when non-optimized compounds are employed in assays, as reported above.

In sum, our method represents a novel application of gene expression data for small molecule—protein interaction prediction, with structural analysis further enriching hits to an unprecedented level in a proteome-scale screen. The success of our proof-of-concept experiments opens the door for a compound-centric drug discovery pipeline that can leverage the relatively small fraction of potentially bioactive compounds that could be of interest for further investigation to become drugs [62]. Interestingly, even relatively weak compounds are able to leave a fingerprint in gene expression correlations. Compared to alternative approaches, our method would be particularly suitable for scanning for targets of newly synthesized scaffolds. We are hopeful that our open source method and predictions might be useful to other labs around the world for identifying new drugs for key proteins involved in various diseases and for better understanding the impact of drug modulation of gene expression. Moreover, our approach represents a new framework for extracting robust correlations from intrinsically noisy gene expression data that reflect the underlying connectivity of the cellular interactome.

## Materials and methods

### Data sources

All predictions and code are open source and available at the supporting website http://sb.cs.cmu.edu/Target2/. A full description of the data used in our analysis can be found in the S1 Text. Briefly, from the NIH LINCS library we extracted gene expression perturbations on 978 “landmark genes” from thousands of small molecule treatment and gene KD experiments in various cell lines. We then used ChEMBL [63], an open large-scale bioactivity database, to identify the LINCS compounds that were FDA approved and had known targets. To construct our validation set we selected the 152 FDA approved compounds that had been tested in at least four distinct LINCS cell lines, and whose known targets were knocked down in the same cell lines. Protein-protein interaction data used in feature construction was extracted from BioGRID [32] and HPRD [64], both of which contain curated sets of physical and genetic interactions. Protein cellular localization data used in feature construction was obtained from the Gene Ontology database [65].

### Extracting and integrating features from different data sources

The notation and symbols that we use in constructing and using the genomic features are described in S6 and S7 Tables. Feature construction is summarized below and is explained in detail in the S1 Text.

#### Direct correlation

The first feature *f*_*cor*_, computes the correlation between the expression profiles resulting from a gene KD and treatment with the small molecule. Since we are considering multiple cells for each molecule/KD, the correlation feature for each molecule *d*, i.e. *f*_*cor*_(*d*,·,·), has a dimension of |*T*_*d*_| × |*C*_*d*_|.

#### Indirect correlation

Information about protein interaction networks may be informative about additional KD experiments that we might expect to be correlated with the small molecule treatment profile. To construct a feature that can utilize this idea we did the following: for each molecule, protein, and cell line we computed *f*_*PC*_(*d*, *g*, *c*), which encodes the fraction of the known binding partners of *g* (i.e. the proteins interacting with *g*) in the top *X* KD experiments correlated with this molecule/cell compared to what is expected based on the degree of that protein (the number of interaction partners—this corrects for hub proteins). We used *X* = 100 here, though 50 and 200 gave similar results. See S1 Text for complete details.

#### Cell selection

While the correlation feature is computed for all cells, it is likely that most drugs are only active in certain cell types and not others (cell lines used in this study are listed in S8 Table). Since the ability to consider the cellular context is one of the major advantages of our method, we added a feature to denote the impact a drug has on a cell line. For each drug/molecule *d* we compute a cell specific feature, *f*_*CS*_(*d*,·), which measures the correlation between the response expression profile and the control (WT) experiments for that cell. We expect a smaller correlation if the drug/molecule is active in this cell, and a larger correlation if it is not.

#### Differential expression

In addition to determining the correlation-based rankings of interacting proteins, we also took their drug-induced differential expression into account. We constructed two features that summarize this information for each protein (see S1 Text for details). These features either encode the average or the max (absolute value) expression level of the interaction partners of the potential target protein.

### Generating structural models for docking

In order to use molecular docking to enrich our random forest predictions, we needed to generate structural models for the genes profiled in LINCS. The union of our top 100 target predictions for the 1680 small molecules profiled in LINCS in at least four cell lines consisted of 3333 unique human genes. We used a python script (available on https://github.com/npabon/generate_gene_models) to mine the PDB for structures of these genes and then select representative crystal structures for each. When multiple structures were available, a representative subset of structures were chosen so as to maximize sequence coverage, minimize structural resolution, and account for structural heterogeneity. Full details of this procedure can be found in the S1 Text.

### Docking procedure

Compounds were docked to representative structures of their predicted targets with smina [37], using default exhaustiveness and a 6 Å buffer to define the box around each potential binding site. Docked poses across predicted binding sites [66] on a given target were compared and the highest scoring pose of each compound was selected for further analyses [36–39] and comparison to other targets/compounds.

### Experimental assays

Full details on all experimental assays involving HRAS, KRAS, CHIP and PDK1 can be found in S1 Text.

## Supporting information

S1 Fig Comparing random forest approaches with a random classifier for predicting known targets of validation compounds. The red arrow indicates the success rate of on-the-fly random forest and the green arrow represents the two-level random forest.
(TIFF)

S2 Fig Receiver operating characteristic (ROC) curve analysis of two-level random forest classification model for predicting the known target of 152 FDA-approved drugs. (TIFF)

S3 Fig Receiver operating characteristic (ROC) curve analysis of structure-enriched target predictions for 53 FDA-approved validation compounds with known target structures. (TIFF)

S4 Fig ZINC compounds weakly disrupt CHIP binding to chaperone peptide as measured by fluorescence polarization. Results are the average and standard error of the mean of two experiments each performed in triplicate.
(TIFF)

S5 Fig CHIP inhibitors prevent ubiquitination by CHIP *in vitro*. (a) Anti-GST western blot showing a lack of substrate ubiquitination in in vitro reactions conducted without CHIP, the GST-Hsc70_395-646_ substrate, or ATP, or where either GST is substituted for the full-length GST-fused substrate or where methylated ubiquitin (mUb) is substituted for human ubiquitin. (b) Anti-GST western blot showing substrate ubiquitination by CHIP in reactions treated with highly ranked (2.1, 2.2) and a low ranked (2.5) compound. (c) Anti-ubiquitin western blot showing total ubiquitination by CHIP in reactions treated with highly ranked (2.1, 2.2) and a low ranked (2.5) compound. (d) Anti-ubiquitin western blot showing ubiquitination by CHIP in in vitro reactions treated with 500 μM of compound 1.7, which was identified as a candidate inhibitor through a pharmacophore-based screen of the ZINC database.
(TIFF)

S6 Fig Predicted CHIP inhibitors prevent ubiquitination of an alternate substrate. (A) Anti-GST western blot showing AT-3 JD substrate ubiquitination by CHIP in reactions treated with compounds. (B) Quantification of all reactions as in A treated with up to 500 μM compound 2.1, 2.2, or 2.6, normalized to ubiquitination by a DMSO treated control (all compounds: N = 4).
(TIFF)

S7 Fig Comparison of gene expression-based and pharmacophore-based virtual screens against CHIP. **HSP90** shows structure of the CHIP (grey)—HSP90 (magenta) interface (PDB ID: 2C2L [49]), indicating the hydrophobic (green spheres) and polar contact (blue surface / dashed lines) pharmacophores used to screen the ZINC database. **Strong binders** show predicted binding modes for compounds 2.1 and 2.2 from the LINCS screen, which showed the strongest FP signal and robust inhibition of CHIP ligases activity. Interestingly, 2.1 and 2.2 are the only predicted hits to make a novel hydrogen bond to CHIP residue Q102, a contact whose importance is not obvious from the cocrystal structure. **Weak binders** show predicted binding modes for compounds 2.3 and 2.4 from the LINCS screen, and compounds 1.1, 1.2, and 1.7 from the ZINC screen, which showed modest FP signal. **Non-binders** show predicted binding modes for non-binding LINCS compounds 2.5 and 2.6, and non-binding ZINC compounds 1.3–1.6.
(TIFF)

S1 Table Compounds in validation set with multiple known protein targets. (DOCX)

S2 Table Compounds in validation set with known protein targets knocked down in 4 or more LINCS cell lines. Parentheses in the rightmost column indicate the predicted ranking of each known target out of over 3000 potential targets.
(DOCX)

S3 Table The cellular localization of successful and unsuccessful drug targets enriched by gene ontology. P-values were computed by intersecting proteins assigned to GO terms listed below with proteins in the sets compared (successful and failed) using the hypergeometric distribution.
(DOCX)

S4 Table Predicted HRAS/KRAS-targeting compounds purchased for experimental validation. ‘Target Rank’ indicates the ranking of HRAS/KRAS in the RF-predicted list of potential targets for each compound. ‘Cpd Rank’ indicates the structure-based ranking of the compound after docking all candidate inhibitors.
(DOCX)

S5 Table Predicted CHIP-targeting compounds purchased for experimental testing. ‘Chip Rank’ indicates the ranking of CHIP in the random-forest predicted list of potential targets for each compound. ‘Cpd Rank’ indicates the structure-based ranking of the compound after docking all candidate inhibitors.
(DOCX)

S6 Table Symbols and notations. (DOCX)

S7 Table Summary of constructed feature sets. Note that different feature sets can have different dimensions (some contain values for each of the cell lines, etc…). The exact dimension and content of each feature set is discussed in the text.
(DOCX)

S8 Table Cell lines included in the validation dataset. The number of drugs, knockdown genes, and control experiment are shown. For a given cell line, we only include drugs that have their target knockdown experiments available in that cell line.
(DOCX)

S1 Text Supplemental methods. (DOCX)

S2 Text Results of testing our random forest classifier on the 123 FDA approved drugs profiled in 4–6 LINCS cell lines after having trained our model on the 29 FDA approved drugs profiled in all 7 LINCS cell lines. The rank of the highest-ranking known target for each compound is listed next to their LINCS ID. We achieve top-100 predictions for 32 drugs, a 26% success rate.
(XLSX)

S3 Text Structural enrichment of random forest predictions for validation hits and comparison with existing methods. Table lists the 63 ‘hits’ from our validation drug set, including their names, LINCS ID and the number of top-100 predicted targets that had structures available in the PDB. The ranking of the known targets for each compound are shown after our genomic random forest target prediction (GEN), and after our structural re-ranking (STR), along with the percentile rankings produced by alternative target prediction methods HTDocking (HTD) and PharmMapper (PHM). STR, HTD, and PHM values of 100 indicate that the structure of the known target either is not known or was not included in the set of potential targets used by the method.
(XLSX)
