# Supplementary figures and images for: Predicting protein targets for drug-like compounds using transcriptomics
Source: PLoS Comput Biol. 2018 Dec 7;14(12):e1006651. doi: 10.1371/journal.pcbi.1006651 (PMC6300300; doi:10.1371/journal.pcbi.1006651)

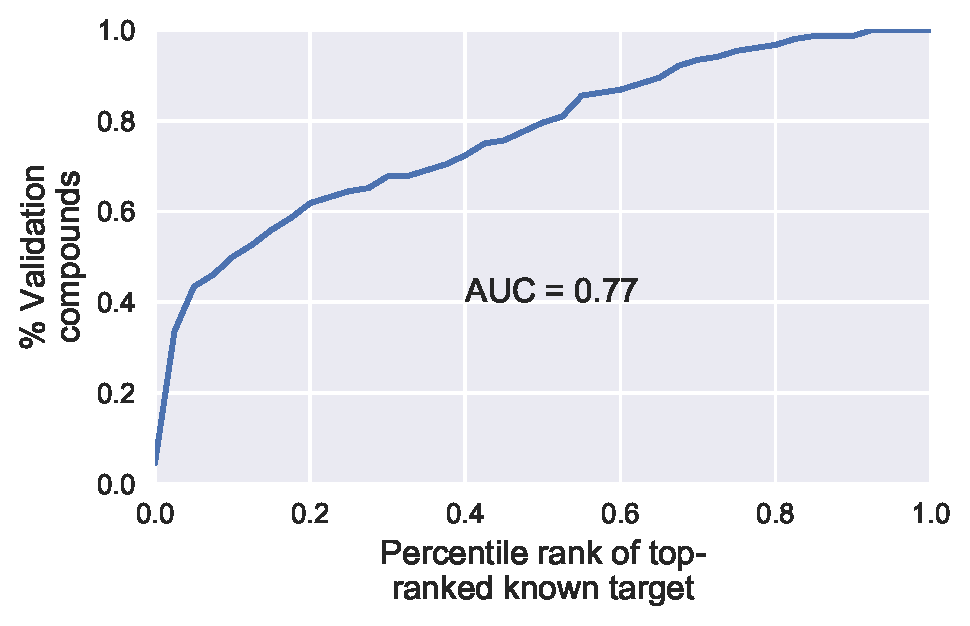

Supplement: S1 Fig — The red arrow indicates the success rate of on-the-fly random forest and the green arrow represents the two-level random forest. (TIFF) [file pcbi.1006651.s001.tiff]

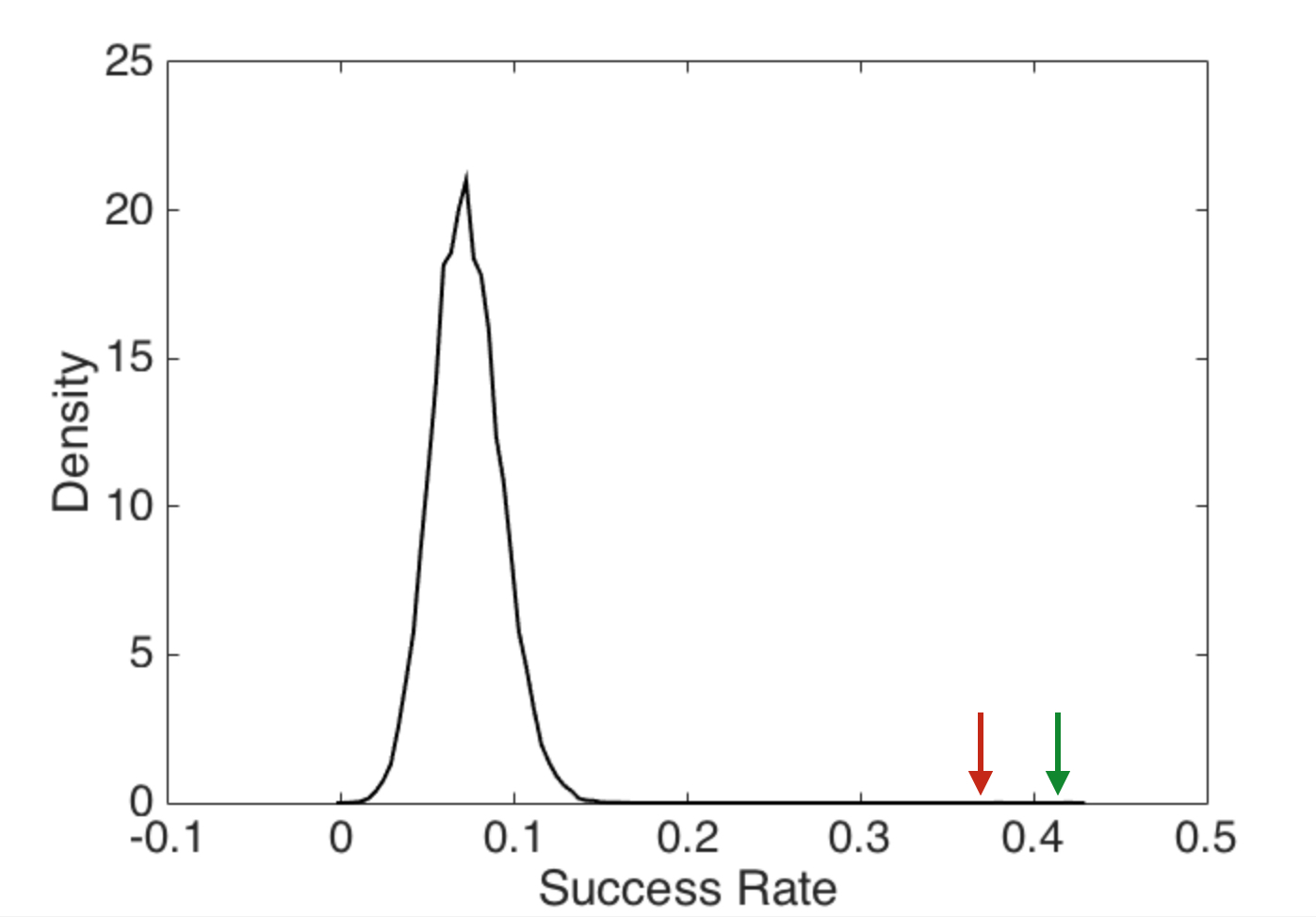

Supplement: S2 Fig — (TIFF) [file pcbi.1006651.s002.tiff]

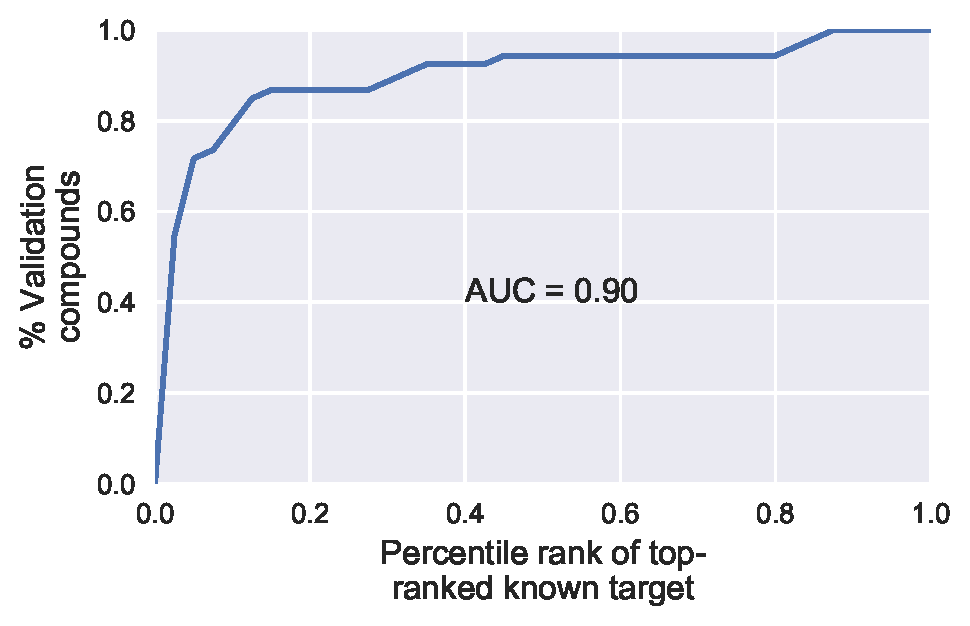

Supplement: S3 Fig — (TIFF) [file pcbi.1006651.s003.tiff]

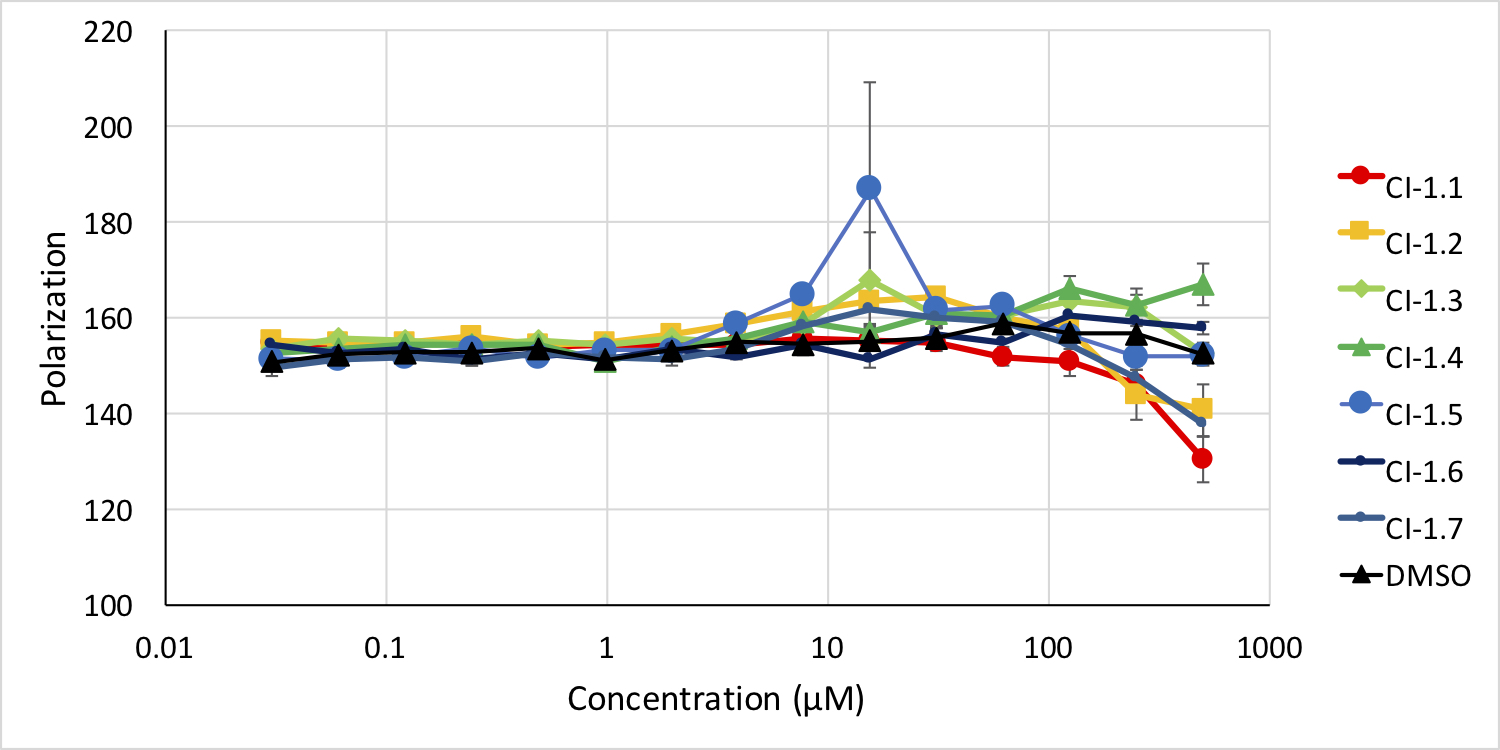

Supplement: S4 Fig — Results are the average and standard error of the mean of two experiments each performed in triplicate. (TIFF) [file pcbi.1006651.s004.tiff]

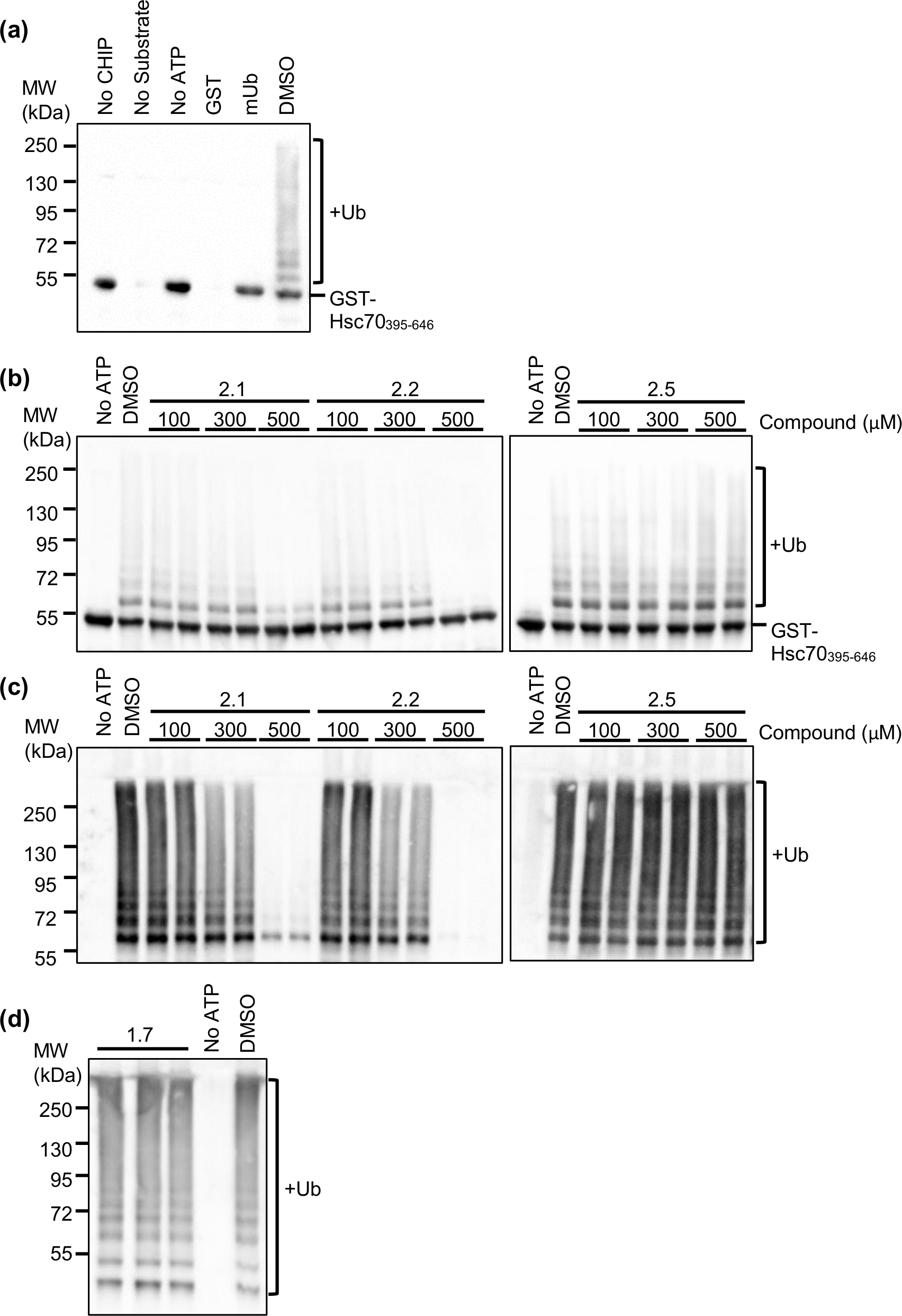

Supplement: S5 Fig — (a) Anti-GST western blot showing a lack of substrate ubiquitination in in vitro reactions conducted without CHIP, the GST-Hsc70395-646 substrate, or ATP, or where either GST is substituted for the full-length GST-fused substrate or where methylated ubiquitin (mUb) is substituted for human ubiquitin. (b) Anti-GST western blot showing substrate ubiquitination by CHIP in reactions treated with highly ranked (2.1, 2.2) and a low ranked (2.5) compound. (c) Anti-ubiquitin western blot showing total ubiquitination by CHIP in reactions treated with highly ranked (2.1, 2.2) and a low ranked (2.5) compound. (d) Anti-ubiquitin western blot showing ubiquitination by CHIP in in vitro reactions treated with 500 μM of compound 1.7, which was identified as a candidate inhibitor through a pharmacophore-based screen of the ZINC database. (TIFF) [file pcbi.1006651.s005.tiff]

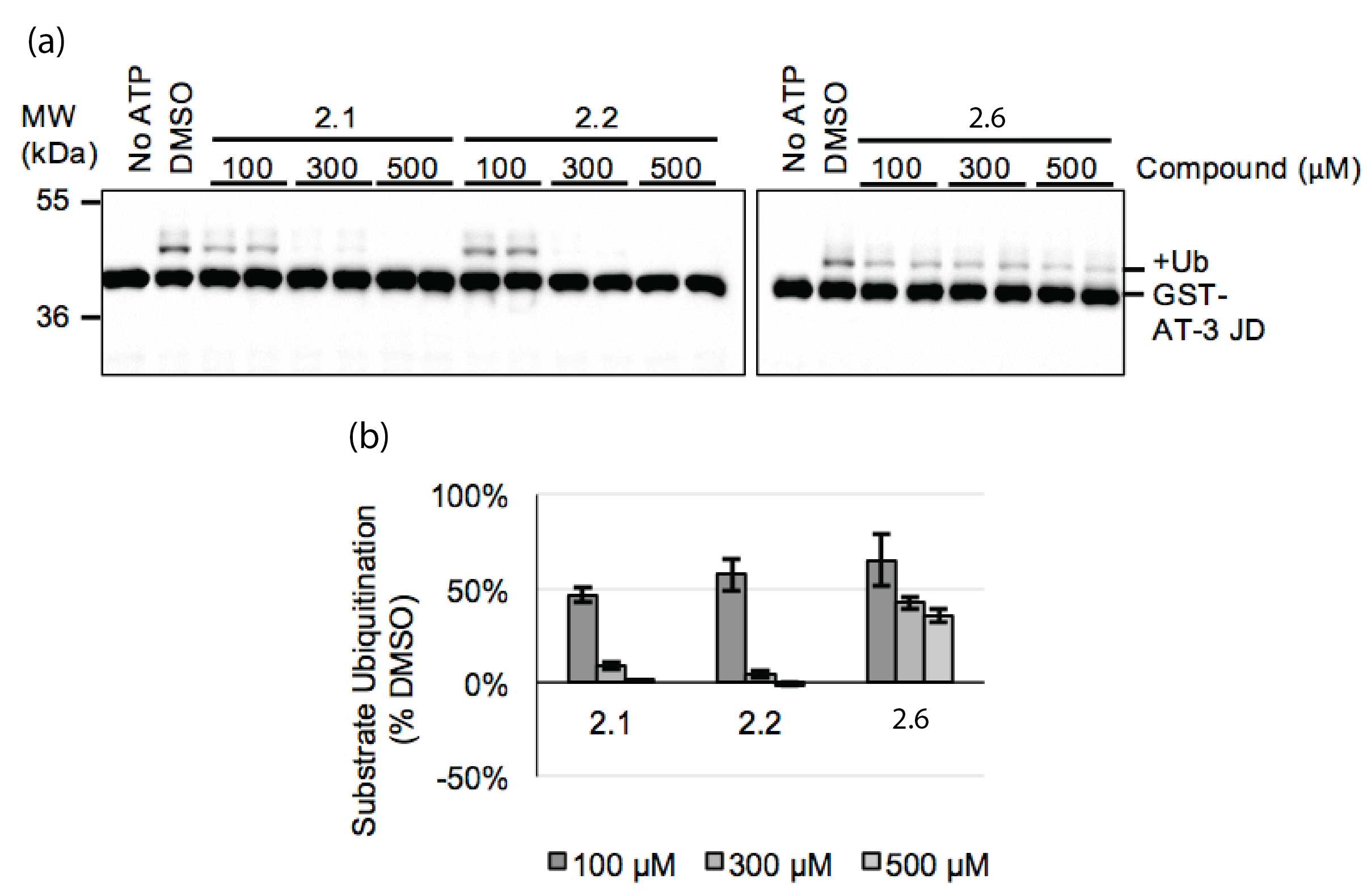

Supplement: S6 Fig — (A) Anti-GST western blot showing AT-3 JD substrate ubiquitination by CHIP in reactions treated with compounds. (B) Quantification of all reactions as in A treated with up to 500 μM compound 2.1, 2.2, or 2.6, normalized to ubiquitination by a DMSO treated control (all compounds: N = 4). (TIFF) [file pcbi.1006651.s006.tiff]

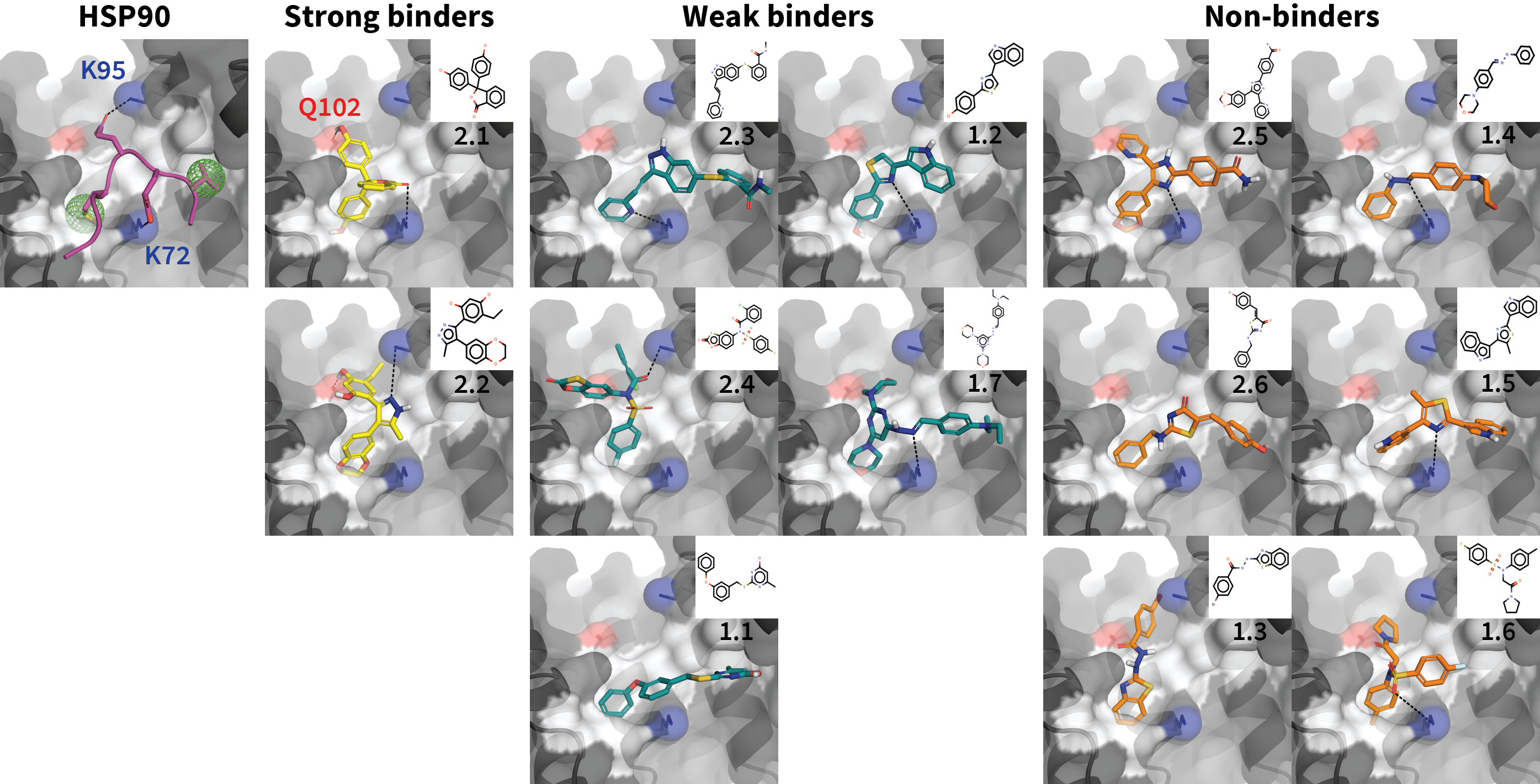

Supplement: S7 Fig — HSP90 shows structure of the CHIP (grey)—HSP90 (magenta) interface (PDB ID: 2C2L [49]), indicating the hydrophobic (green spheres) and polar contact (blue surface / dashed lines) pharmacophores used to screen the ZINC database. Strong binders show predicted binding modes for compounds 2.1 and 2.2 from the LINCS screen, which showed the strongest FP signal and robust inhibition of CHIP ligases activity. Interestingly, 2.1 and 2.2 are the only predicted hits to make a novel hydrogen bond to CHIP residue Q102, a contact whose importance is not obvious from the cocrystal structure. Weak binders show predicted binding modes for compounds 2.3 and 2.4 from the LINCS screen, and compounds 1.1, 1.2, and 1.7 from the ZINC screen, which showed modest FP signal. Non-binders show predicted binding modes for non-binding LINCS compounds 2.5 and 2.6, and non-binding ZINC compounds 1.3–1.6. (TIFF) [file pcbi.1006651.s007.tiff]
