# Supplementary material for: Predicting protein targets for drug-like compounds using transcriptomics
Source: PLoS Comput Biol. 2018 Dec 7;14(12):e1006651. doi: 10.1371/journal.pcbi.1006651 (PMC6300300; doi:10.1371/journal.pcbi.1006651)
Supplement: S1 Table — (DOCX) [file pcbi.1006651.s008.docx]

**Table S1. Compounds in validation set with multiple known protein targets.**

| **NAME** | **ID** | **KNOWN TARGETS** |
| --- | --- | --- |
| clofarabine | BRD-A82371568 | POLE, POLA1 |
| glibenclamide | BRD-K36927236 | KCNJ11, ABCC8 |
| vincristine | BRD-A76528577 | TUBA1C, TUBA3D, TUBB4, TUBB2B, TUBA1B, TUBA4A, TUBB8, TUBB6, TUBB2C, TUBA3E, TUBB2A, TUBA1A, TUBA3C |
| etodolac | BRD-A16998493 | PTGS2, COX2 |
| digoxin | BRD-A75144621 | ATP1A1, ATP1B3, ATP1A2, ATP1B1, ATP1A4, ATP1B2, FXYD2, ATP1A3 |
| gemcitabine | BRD-K15108141 | POLE, POLA1 |
| paclitaxel | BRD-A23723433 | TUBA1C, TUBA3D, TUBB4, TUBB2B, TUBA1B, TUBA4A, TUBB8, TUBB6, TUBB2C, TUBA3E, TUBB2A, TUBA1A, TUBA3C |
| vincristine | BRD-K82109576 | TUBA1C, TUBA3D, TUBB4, TUBB2B, TUBA1B, TUBA4A, TUBB8, TUBB6, TUBB2C, TUBA3E, TUBB2A, TUBA1A, TUBA3C |
| fludarabine | BRD-K72238567 | POLE, POLA1 |
| bortezomib | BRD-K88510285 | PSMA4, PSMB9, PSMA3, PSMA6, PSMB7, PSMA7, PSMA2, PSMA5, PSMB5, PSMA1, PSMB3, PSMA8, PSMB10, PSMB1, PSMB4, PSMB11 |
| celecoxib | BRD-K02637541 | PTGS2, COX2 |
| dasatinib | BRD-K49328571 | YES1, BLK, FRK, LCK |
| sibutramine | BRD-A23359898 | SLC6A5, SLC6A2, NAT1, NET1 |
| cytarabine | BRD-K33106058 | POLE, POLA1 |
| vinorelbine | BRD-K10916986 | TUBA1C, TUBA3D, TUBB4, TUBB2B, TUBA1B, TUBA4A, TUBB8, TUBB6, TUBB2C, TUBA3E, TUBB2A, TUBA1A, TUBA3C |
| alitretinoin | BRD-K35483542 | RARA, RARG, RXRA |
| digoxin | BRD-K23478508 | ATP1A1, ATP1B3, ATP1A2, ATP1B1, ATP1A4, ATP1B2, FXYD2, ATP1A3 |
| nabumetone | BRD-K65146499 | PTGS2, COX2 |
| digoxin | BRD-A94756469 | ATP1A1, ATP1B3, ATP1A2, ATP1B1, ATP1A4, ATP1B2, FXYD2, ATP1A3 |
| paclitaxel | BRD-A28746609 | TUBA1C, TUBA3D, TUBB4, TUBB2B, TUBA1B, TUBA4A, TUBB8, TUBB6, TUBB2C, TUBA3E, TUBB2A, TUBA1A, TUBA3C |
| vinorelbine | BRD-K97514127 | TUBA1C, TUBA3D, TUBB4, TUBB2B, TUBA1B, TUBA4A, TUBB8, TUBB6, TUBB2C, TUBA3E, TUBB2A, TUBA1A, TUBA3C |
| vincristine | BRD-A60414806 | TUBA1C, TUBA3D, TUBB4, TUBB2B, TUBA1B, TUBA4A, TUBB8, TUBB6, TUBB2C, TUBA3E, TUBB2A, TUBA1A, TUBA3C |
| vinblastine | BRD-A22783572 | TUBA1C, TUBA3D, TUBB4, TUBB2B, TUBA1B, TUBA4A, TUBB8, TUBB6, TUBB2C, TUBA3E, TUBB2A, TUBA1A, TUBA3C |
| glimepiride | BRD-K34776109 | KCNJ11, ABCC8 |
| vinblastine | BRD-A55594068 | TUBA1C, TUBA3D, TUBB4, TUBB2B, TUBA1B, TUBA4A, TUBB8, TUBB6, TUBB2C, TUBA3E, TUBB2A, TUBA1A, TUBA3C |
| vinorelbine | BRD-M30523314 | TUBA1C, TUBA3D, TUBB4, TUBB2B, TUBA1B, TUBA4A, TUBB8, TUBB6, TUBB2C, TUBA3E, TUBB2A, TUBA1A, TUBA3C |
| valdecoxib | BRD-K12994359 | PTGS2, COX2 |
| alprazolam | BRD-K32398298 | GABRP, GABRQ, GABRA4, GABRB1, GABRE, GABRB2, GABRG1 |
| pentobarbital | BRD-A44448661 | GABRP, GABRQ, GABRA4, GABRB1, GABRE, GABRB2, GABRG1 |
| glipizide | BRD-K12219985 | KCNJ11, ABCC8 |
| tolazamide | BRD-K32164935 | KCNJ11, ABCC8 |
| etodolac | BRD-K99260425 | PTGS2, COX2 |
| digitoxin | BRD-A93236127 | ATP1A1, ATP1B3, ATP1A2, ATP1B1, ATP1A4, ATP1B2, FXYD2, ATP1A3 |
