# Supplementary material for: Predicting protein targets for drug-like compounds using transcriptomics
Source: PLoS Comput Biol. 2018 Dec 7;14(12):e1006651. doi: 10.1371/journal.pcbi.1006651 (PMC6300300; doi:10.1371/journal.pcbi.1006651)
Supplement: S2 Table — Parentheses in the rightmost column indicate the predicted ranking of each known target out of over 3000 potential targets. (DOCX) [file pcbi.1006651.s009.docx]

**Table S2. Compounds in validation set with known protein targets knocked down in 4 or more LINCS cell lines.** Parentheses in the rightmost column indicate the predicted ranking of each known target out of over 3000 potential targets.

| **NAME** | **ID** | **RANKED TARGETS** |
| --- | --- | --- |
| vincristine | BRD-A76528577 | TUBA1A (17), TUBB6 (159) |
| paclitaxel | BRD-A23723433 | TUBA1A (141), TUBB6 (86) |
| vincristine | BRD-K82109576 | TUBA1A (74), TUBB2C (1656), TUBB6 (288), TUBB2A (108) |
| bortezomib | BRD-K88510285 | PSMB7 (86), PSMA3 (27), PSMA5 (70), PSMB5 (22), PSMA1 (40), PSMA8 (195), PSMB10 (424) |
| dasatinib | BRD-K49328571 | YES1 (1039), LCK (649) |
| sibutramine | BRD-A23359898 | NAT1 (1855), NET1 (2044) |
| vinorelbine | BRD-K10916986 | TUBA1A (14), TUBB6 (95) |
| alitretinoin | BRD-K35483542 | RARA (2616), RARG (126), RXRA (175) |
| paclitaxel | BRD-A28746609 | TUBA1A (1), TUBB6 (116) |
| vinorelbine | BRD-K97514127 | TUBA1A (48), TUBB2C (2977), TUBB6 (264), TUBB2A (450) |
| vincristine | BRD-A60414806 | TUBA1A (9), TUBB6 (23) |
| vinblastine | BRD-A22783572 | TUBA1A (1), TUBB6 (46) |
| vinblastine | BRD-A55594068 | TUBA1A (6), TUBB6 (137) |
| vinorelbine | BRD-M30523314 | TUBA1A (152), TUBB2C (1670), TUBB6 (344), TUBB2A (419) |
| alprazolam | BRD-K32398298 | GABRP (2285), GABRG1 (2284) |
| pentobarbital | BRD-A44448661 | GABRP (1377), GABRG1 (2577) |
