# Supplementary material for: Predicting protein targets for drug-like compounds using transcriptomics
Source: PLoS Comput Biol. 2018 Dec 7;14(12):e1006651. doi: 10.1371/journal.pcbi.1006651 (PMC6300300; doi:10.1371/journal.pcbi.1006651)
Supplement: S3 Table — P-values were computed by intersecting proteins assigned to GO terms listed below with proteins in the sets compared (successful and failed) using the hypergeometric distribution. (DOCX) [file pcbi.1006651.s010.docx]

**Table S3. The cellular localization of successful and unsuccessful drug targets enriched by gene ontology.** P-values were computed by intersecting proteins assigned to GO terms listed below with proteins in the sets compared (successful and failed) using the hypergeometric distribution.

|  | **Cellular Component** | **p-value** |
| --- | --- | --- |
| **Successful Targets** | proteasome core complex | 7.81E-37 |
|  | proteasome core | 1.10E-28 |
|  | proteasome alpha-subunit | 5.68E-18 |
|  | cytosol | 7.53E-12 |
|  | protein complex | 1.88E-11 |
| **Failed Targets** | transmembrane transporter complex | 7.77E-15 |
|  | sodium-exchanging ATPase complex | 4.42E-14 |
|  | cation-transporting ATPase complex | 8.74E-13 |
|  | plasma membrane part | 2.19E-11 |
|  | chloride channel complex | 2.33E-09 |
