# Supplementary material for: Predicting protein targets for drug-like compounds using transcriptomics
Source: PLoS Comput Biol. 2018 Dec 7;14(12):e1006651. doi: 10.1371/journal.pcbi.1006651 (PMC6300300; doi:10.1371/journal.pcbi.1006651)
Supplement: S4 Table — ‘Target Rank’ indicates the ranking of HRAS/KRAS in the RF-predicted list of potential targets for each compound. ‘Cpd Rank’ indicates the structure-based ranking of the compound after docking all candidate inhibitors. (DOCX) [file pcbi.1006651.s011.docx]

**Table S4. Predicted HRAS/KRAS-targeting compounds purchased for experimental validation**. ‘Target Rank’ indicates the ranking of HRAS/KRAS in the RF-predicted list of potential targets for each compound. ‘Cpd Rank’ indicates the structure-based ranking of the compound after docking all candidate inhibitors.

| **Target** | **Name** | **ID** | **Target Rank** | **Cpd Rank** |
| --- | --- | --- | --- | --- |
| HRAS | BRD-A18725729 | BRD-A18725729 | 90 | 52 |
|  | BRD-K00954209 | BRD-K00954209 | 73 | 1 |
|  | BRD-K95858622 | BRD-K95858622 | 92 | 34 |
|  | mefloquine | BRD-K40645748 | 56 | 34 |
|  | procaterol | BRD-A22684332 | 99 | 70 |
|  | RS-39604 | BRD-K20742498 | 21 | 81 |
| KRAS | KM_00799 | BRD_K87375115 | 6 | 84 |
|  | phloretin | BRD_K15563106 | 65 | 3 |
|  | zardaverine | BRD_K37561857 | 34 | 67 |
|  | BRD_K85275009 | BRD_K85275009 | 1 | 80 |
|  | amodiaquine | BRD_K91290917 | 35 | 32 |
