# Supplementary material for: Predicting protein targets for drug-like compounds using transcriptomics
Source: PLoS Comput Biol. 2018 Dec 7;14(12):e1006651. doi: 10.1371/journal.pcbi.1006651 (PMC6300300; doi:10.1371/journal.pcbi.1006651)
Supplement: S5 Table — ‘Chip Rank’ indicates the ranking of CHIP in the random-forest predicted list of potential targets for each compound. ‘Cpd Rank’ indicates the structure-based ranking of the compound after docking all candidate inhibitors. (DOCX) [file pcbi.1006651.s012.docx]

**Table S5. Predicted CHIP-targeting compounds purchased for experimental testing**. ‘Chip Rank’ indicates the ranking of CHIP in the random-forest predicted list of potential targets for each compound. ‘Cpd Rank’ indicates the structure-based ranking of the compound after docking all candidate inhibitors.

| Cpd # | Name | ID | CHIP Rank | Cpd  Rank |
| --- | --- | --- | --- | --- |
| 2.1 | phenolphthalein | BRD_K19227686 | 2 | 22 |
| 2.2 | HSP90_inhibitor | BRD_K65503129 | 2 | 4 |
| 2.3 | axitinib | BRD_K29905972 | 8 | 13 |
| 2.4 | BRD_K59556282 | BRD_K59556282 | 11 | 92 |
| 2.5 | SB_431542 | BRD_K67298865 | 34 | 17 |
| 2.6 | MW_STK33_2B | BRD_K78930611 | 51 | 16 |
