# Supplementary material for: Predicting protein targets for drug-like compounds using transcriptomics
Source: PLoS Comput Biol. 2018 Dec 7;14(12):e1006651. doi: 10.1371/journal.pcbi.1006651 (PMC6300300; doi:10.1371/journal.pcbi.1006651)
Supplement: S6 Table — (DOCX) [file pcbi.1006651.s013.docx]

**Table S6. Symbols and notations.**

| **Symbol** | **Meaning** |
| --- | --- |
| *d* | Index for a drug |
| *c* | Index for a cell line |
| *g* | Index for a gene |
| *N_D_* | Total number of genes |
| *N_C_* | Total number of cell lines |
| *C_d_* | The set of cell line indeces for drug *d* |
| *P_d_* | The set of protein target indeces for drug *d* |
| *G_c_* | The set of knockdown gene indeces for cell line *c* |
| *T_d_* | The intersection of knockdown gene indeces *G_c_* for all cell lines in *C_d_* |
| *N_dc_* | Number of experiments for applying drug *d* to cell line *c* |
| *N_gc_* | Number of experiments for knocking down gene *g* in cell line *c* |
| *N_g_* | Neighbors, or protein-protein interaction partners, of gene *g* |
| *Δ* | Drug-response data |
| *Γ* | Gene-knockdown data |
| *Ψ* | Control data |
| *Ω* | Full feature data |
| *X_d_* | Training data derived from drug *d* |
| *y_d_* | Training label derived from drug *d* |
| *ν_d_* | Negative (non-target) genes for drug *d* |
