# Supplementary material for: Predicting protein targets for drug-like compounds using transcriptomics
Source: PLoS Comput Biol. 2018 Dec 7;14(12):e1006651. doi: 10.1371/journal.pcbi.1006651 (PMC6300300; doi:10.1371/journal.pcbi.1006651)
Supplement: S7 Table — Note that different feature sets can have different dimensions (some contain values for each of the cell lines, etc…). The exact dimension and content of each feature set is discussed in the text. (DOCX) [file pcbi.1006651.s014.docx]

**Table S7. Summary of constructed feature sets.** Note that different feature sets can have different dimensions (some contain values for each of the cell lines, etc…). The exact dimension and content of each feature set is discussed in the text.

| **Feature Name** | **Symbol** | **Meaning** |
| --- | --- | --- |
| Direct Correlation | *f_cor_* | Correlation between a drug treatment experiment and a gene knockdown experiment |
| Indirect Correlation | *f_PC_* | Fraction of the known binding partners of a gene in the top *X* correlated knockdown experiments |
| Cell Selection | *f_CS_* | Correlation between a drug treatment experiment and the control experiment for the cell line |
| PPI Expression | *f_PE_* | The average or the max (absolute value) expression for the known binding partners of a gene |
