# Supplementary material for: Predicting protein targets for drug-like compounds using transcriptomics
Source: PLoS Comput Biol. 2018 Dec 7;14(12):e1006651. doi: 10.1371/journal.pcbi.1006651 (PMC6300300; doi:10.1371/journal.pcbi.1006651)
Supplement: S8 Table — The number of drugs, knockdown genes, and control experiment are shown. For a given cell line, we only include drugs that have their target knockdown experiments available in that cell line. (DOCX) [file pcbi.1006651.s015.docx]

**Table S8.** **Cell lines included in the validation dataset.** The number of drugs, knockdown genes, and control experiment are shown. For a given cell line, we only include drugs that have their target knockdown experiments available in that cell line.

| **Cell Line** | **Drugs** | **Knockdowns** | **Controls** |
| --- | --- | --- | --- |
| A549 | 188 | 11947 | 52 |
| MCF7 | 180 | 12031 | 54 |
| VCAP | 175 | 13225 | 56 |
| HA1E | 172 | 11968 | 53 |
| A375 | 143 | 11696 | 58 |
| HCC515 | 129 | 7828 | 52 |
| HT19 | 96 | 10185 | 52 |
