# Supplementary material for: Predicting protein targets for drug-like compounds using transcriptomics
Source: PLoS Comput Biol. 2018 Dec 7;14(12):e1006651. doi: 10.1371/journal.pcbi.1006651 (PMC6300300; doi:10.1371/journal.pcbi.1006651)
Supplement: S1 Text — (DOCX) [file pcbi.1006651.s016.docx]

**Supplemental Information:**

**Predicting protein targets for drug-like compounds using transcriptomics**

Nicolas A. Pabon1, (npabon@pitt.edu), Yan Xia2, (xiayan0118@gmail.com), Samuel K. Estabrooks3 (ske12@pitt.edu), Zhaofeng Ye4 (zhy44@pitt.edu), Amanda K. Herbrand5 (amanda.herbrand@stud.uni-frankfurt.de), Evelyn Süß5 (suess@med.uni-frankfurt.de), Ricardo M. Biondi5 (dabiondi@yahoo.co.uk), Victoria A. Assimon6 (vaa@umich.edu), Jason E. Gestwicki6 (Jason.gestwicki@ucsf.edu), Jeffrey L. Brodsky3 (jbrodsky@pitt.edu), Carlos J. Camacho1,* (ccamacho@pitt.edu), and Ziv Bar-Joseph2 (zivbj@cs.cmu.edu)

1 Department of Computational and Systems Biology, University of Pittsburgh , Pittsburgh, PA 15213

2 Machine Learning Department, School of Computer Science, Carnegie Mellon University, 15213

3 Department of Biological Sciences, University of Pittsburgh, Pittsburgh, PA 15260

4 School of Medicine, Tsinghua University, Beijing, China 100084

5 Department of Internal Medicine I, Universitätsklinikum Frankfurt, 60590 Frankfurt, Germany

6 Department of Pharmaceutical Chemistry, University of California, San Francisco, San Francisco, CA 94158

These two authors contributed equally

* To whom correspondence should be addressed

**Corresponding Author:**

Carlos Camacho

3077 Biomedical Science Tower 3

3501 Fifth Avenue

Pittsburgh, PA 15260

412-648-7776, Fax: 412-648-3163

E-mail: [ccamacho@pitt.edu](mailto:ccamacho@pitt.edu)

**Availability of data and materials**:

All predictions and code are open source and available at the supporting website http://sb.cs.cmu.edu/Target2/.

**Supplemental Methods**

**Data sources**

*LINCS:* LINCS is an NIH program that generates and curates gene expression profiles across multiple cell lines and perturbation types at a massive scale. To date, LINCS has generated millions of gene expression profiles (over 150 gigabytes of data) containing small-molecules and genetic gain- (cDNA) and loss-of-function (sh-RNA) constructs across multiple cell types. Specifically, the LINCS dataset contains experiments profiling the effects of 20,143 small-molecule compounds (including known drugs) and 22,119 genetic constructs for over-expressing or knocking-down genes performed in 18 different cell types selected from diverse lineages which span established cancer cell lines, immortalized (but not transformed) primary cells, and both cycling and quiescent cells.

The gene expression profiles were measured using a bead-based assay termed the L1000 assay[[1]](#footnote-1). To increase throughput and save costs, this assay only profiles a set of 978 so-called “landmark genes” and the expression values of other genes can be computationally imputed from this set. Note however, that in our analysis we do not rely on such imputation and our methods only need to use the values for the measured genes. In our analysis we used level-4 signature values (containing z-scores for each gene in each experiment based on repeats relative to population control). Data processing of LINCS was done using the l1ktool.[[2]](#footnote-2)

*ChEMBL:* To obtain a list of known targets for the drugs in our validation set we used ChEMBL, an open large-scale bioactivity database [2]. We retrieved the records of all FDA-approved drugs using the ChEMBL web service API[[3]](#footnote-3). These records contain the designed targets for the drugs along with their synonyms (alternate names) and unique chemical IDs. We used this information to cross-reference these drugs with those in LINCS.

*Protein-protein interaction and gene ontology:* We obtained PPI information for our feature sets from BioGRID [3] and HPRD [4], both of which contain curated sets of physical and genetic interactions. We retrieved all the records corresponding to protein-protein interactions (PPI) from these data sources and converted them to an adjacency list representation. We obtained the cellular localization of proteins from the Gene Ontology database [5]. We relied on prior analysis [6] to assign the location of for each protein as either “intracellular” (inside of cell) or “extracellular” (outside of cell).

**Extracting experiments from LINCS**

After determining the subsets of small molecules and cell lines, we obtained the associated experiment identifiers known as “distil IDs” from LINCS meta- information. We included only the reproducible distil IDs known as “Gold” IDs. We then extracted the corresponding signature values from LINCS using the L1000 Analysis Tools (l1ktools)[[4]](#footnote-4). We only extracted the signature values of the 978 “landmark" genes because their expression was directly measured, whereas the values of other genes were imputed from the data of these landmark genes.

*Drug response experiments*

There exist multiple experiments (distil IDs) corresponding to a combination of drug *d* and cell line *c* (applying drug *d* to cell line *c*). Denote the *Ndc* as the number of experiments for the combination *d,c*. We extracted a matrix of signature values of size 978 *Ndc* (number of landmark genes number of experiments) per combination. We next took the median of signature values across different experiments, and obtained a 987 1 signature vector per combination. The overall drug-response data Δ**,** therefore, is implemented as a MATLAB structure with D = 152 entries, each containing the following fields.

**name**: (string)

**cells**: ( 1 string array)

**signature**: (978 )

where is the unique internal identifier of a small molecule *d* in LINCS. contains the expression values of drug *d* across *Cd* different cell lines. The field contains cell line names corresponding to the column of .

*Gene knockdown experiments*

We follow a similar protocol to extract the signature values of gene knockdown experiments. Denote *Ngc* as the number of experiments for the combination of gene *g* and cell line *c* (knocking down gene *g* in cell line *c*). Then, for each combination of *g* and *c* we extracted signature values of size 978 *Ngc*. After taking the medians across different experiments, we obtain a 978 1 vector per combination. The overall gene knockdown data Γ has *C* = 7 entries and each entry contains the following fields:

**name:** (string)

**genes:** ( 1 string array)

**signature:** (978 )

where is the name of the cell line indexed by *c*. contains the signature values of the knockdown of genes in cell line *c*. The field is a subset of gene symbols corresponding to the column identifiers of under the HGNC naming scheme.

*Control experiments*

We also extracted the signatures of control experiments. The signature values for each cell line were extracted and we obtained a 978 1 vector after taking the medians. We denote the overall control experiment data as Ψ. Ψ is of size 978 *C* and implemented with the following format:

**name:** (string)

**control:** (978 1)

where is the signature column vector for a cell line c.

**Building a validation dataset from LINCS**

We used ChEMBL to retrieve the reported targets and other meta-information of all FDA-approved drugs, and then cross referenced these drugs with the small molecules profiled in LINCS using their primary product names, synonyms, canonical SMILES strings and standard InChIKey. Based on this analysis we identified 1031 out of approximately 1300 FDA-approved drugs reported in LINCS. However, most of these drugs were profiled in only one or very few cell lines, which meant that relatively little response data was available for them. We thus further reduced this set to 152 drugs profiled in at least 4 cell lines (Table 2) and used these drugs and their known targets as the positive training set. Table S8 lists the number of drugs and knockdown experiments available for the seven most abundant cell lines in terms of known targets profiled that we used in our analysis.

**Extracting and integrating features from different data sources**

The notation and symbols that we use in constructing and using the genomic features are described in Table S6 and Table S7. Feature construction is summarized below.

*Direct correlation:* The first feature ,computes the correlation between the expression profiles resulting from a gene knockdown and treatment with the small molecule. The correlation feature, denoted as *fcor*, is constructed as follows:

- For each drug *d* in Δ ():

- Denote *Td* as the intersection of gene symbol indices for cells in *Cd*:

- Obtain the knockdown signature values of *Td* from Γ. Denote this data matrix as , which is of size 978 , where for each cell line in *Cd* there is a signature matrix of size 978 .

- Compute the Pearson's correlation between (978 ) and ( 978 ). Specifically, for each cell line , we compute the correlation between and , and obtain a correlation vector of size . This is the correlation between the responses of the cells to the drug treatment and their response to the gene knockdown. Each entry in this vector is the correlation of 978 landmark genes of the drug *d* in one cell line () and a knockdown of gene *g* in the same cell line (). In other words, if we collect these correlation vectors for all cell lines in *Cd* and denote the overall correlation feature as *fcor*:

The correlation feature for one drug *d*, , has a dimension of .

*Indirect correlation:* Information about protein interaction networks may be informative about additional knockdown experiments that we might expect to be correlated with the small molecule treatment profile. To construct a feature that can utilize this idea we did the following: for each molecule, protein, and cell line we computed , which encodes the fraction of the known binding partners of *g* (i.e. the proteins interacting with *g*) in the top *X* knockdown experiments correlated with this molecule/cell compared to what is expected based on the degree of that protein (the number of interaction partners - this corrects for hub proteins). We used *X* = 100 here, though 50 and 200 gave similar results.

The indirect correlation score is constructed as follows:

- For each drug *d* in Δ ():

- Obtain *Td*, as defined above.

- For each cell line *c* in *Cd*:

- Sort *Td* in descending order using the correlation values

- Denote the sorted gene symbol indices for cell line *c* as

- For each knockdown gene *g* in *Td*:

- Obtain the set of neighbor gene symbol indices from the PPI adjacency list, and denote it as *Ng*.

- Compute *fPC* as:

has the same dimension as ( ). It reflects the fraction of gene *g*'s binding partners that are more correlated with drug *d* in the context of cell line *c*. We use 50 as the pseudo-count to penalize hub proteins, which have substantially more neighbors than others.

*Cell selection:* While the correlation feature is computed for all cells, it is likely that most drugs are only active in certain cell types and not others. Since the ability to consider the cellular context is one of the major advantages of our method we added a feature to denote the impact a drug has on a cell line. For each drug/molecule *d* we compute a cell specific feature, , which measures the correlation between the response expression profile and the control (WT) experiments for that cell. We expect a smaller correlation if the drug/molecule is active in this cell, and a larger correlation if it is not. The cell selection feature is calculated

*Differential expression:* In addition to determining the correlation-based rankings of interacting proteins, we also took their drug-induced differential expression into account. We constructed two features that summarize this information for each protein. These features either encode the average or the max (absolute value) expression level of the interaction partners of the potential target protein.

We compute two types of PPI expression scores, denoted as and , as follows:

- For each drug *d* in Δ ():

- For each knockdown gene *g* in *Td*:

- Obtain *Ng*, as above (the list of neighbors, or interaction partners, of *g*)

- For each cell line *c* in *Cd*:

- Find the set of signature values for the neighbors of *g*, (size 1)

- Compute the two PPI expression scores as:

*Feature data structure*

We combined the features for all drugs in a MATLAB structure Ω. Ω has *D* entries, and each entry Ω(d) has the following fields:

**name:** (string)

**targets:**  (protein targets for *d)*

**cells:** ( 1 string array)

**genes:**  (common genes across *Gc*)

**correlation:**  ( )

**PPI correlation:**  ( )

**max PPI expression:**  ( )

**avg PPI expression:**  ( )

**cell selection:**  ( 1)

There are a total of *D* = 152 drugs in Ω, and the number of drugs with different values of are summarized in Table 2.

**Subcellular Localization Assignment**

We obtained the cellular localization of genes from the Gene Ontology Consortium. The GO database provides web services to query genes in terms of their associated biological processes, cellular components and molecular functions in a species-independent manner[[5]](#footnote-5). We further assign the locations as either “intracellular” (inside of cell) or “extracellular” (outside of cell). The detailed assignments are shown in Table S3.

**Classification procedure**

*Criterion of successful classification*

Due to the intrinsic noise from the data, we define a successful classification for a drug if any of its correct targets is enriched into the top *K* ranked genes, where *K* can be either 50 or 100.

*Analysis of feature importance*

The evaluation of single features was performed using the drugs that have been applied on all seven cell lines. There are 29 of these drugs from Ω. We sort (descendingly) the common genes *Td* for a drug *d* and cell line *c* using an individual feature , where is either or . Denote as the ranking of a gene in the context of cell line *c*. Then, we define the overall ranking of a gene, , to be the best ranking across all seven cell lines: for .

*Constructing training dataset*

Next, we wish to learn and evaluate classifiers that predict drug targets using all features from the feature dataset Ω. We first construct a training data set (design matrix *X* and its associated labels *y*) from the feature dataset Ω.

For each drug *d* in Ω, we select the rows corresponding to the targets in *Pd* from the other feature matrices and concatenate them into a row vector. The same cell selection vector is appended to every row of targets. These rows are assigned with a positive label 1. We then randomly sampled 100 non-target genes (denoted as ) and construct the row vectors the same way as the target genes, and these rows are assigned with a negative label 0. In other words, the training matrix and label vector constructed from a drug *d* are of the following format:

;

where , the total number of targets for drug *d*. Therefore, the training matrix *Xd* for drug d is of size , and label vector has length .

**Extending Random forests to Drugs with Missing Features**

Since our goal here is to predict targets for as many small molecule as possible, we did not want to restrict our analysis to molecules that were only profiled in a large number of cell lines. As noted above, requiring at least seven cell lines reduces the number of known drugs that can be evaluated from 152 to 29 and leads to a similar reduction in the number of novel small molecules that can be evaluated. Thus, it is highly desirable that our classifiers can handle missing data (i.e., cells for which experiments were not performed). To this end, we developed two distinct methods to deal with different compound-specific cell line combinations and extended the random forest [7, 8] model so that can handle molecules profiled in less than seven (but more than four) cell types.

In the first method we simply build the random forest “on-the-fly”. For a given drug *i*, we iterate through all other drugs in and test if a drug *d* was profiled in at least all cells which drug *i* was profiled in. In other words, we test if and if so we extract the features of corresponding cell lines in *Ci* from and include them in the training data. After we include data for all compatible drugs we can use the training data to train and apply a random forest for the given drug *i*. We note that for any drug in , there are at least 28 compatible drugs because 29 drugs have been applied to all seven cell lines. However, the main disadvantage of this method is that we need to train separate random forest for every test drug.

In the second method we perform a “two-level” random forest construction process. Here, in addition to the standard step of selecting a (random) subset of the features for each of the trees in the forest we included a step that selected a (random) subset of cells for each of the trees. Specifically, in the first step, we randomly sample four cell lines from the seven total cell lines (denoted as *Ci*). In the second step, we find all drugs such that , extract their features, and use them to train that tree. We repeat this process 3500 times, such that each combination of four cell lines is expected to have roughly 100 trees (). To apply this two-level random forest to a test drug *t* with cell line profile *Ct*, we select from the forest those decision trees *i* for which and use them to predict the targets for *t*. Note that unlike the on-the-fly method above, here we only need to train one forest for the entire prediction task.

**Generating structural models for docking**

In order to use molecular docking to enrich of our random forest predictions, we needed to generate structural models for the genes profiled in LINCS. The union of our top 100 target predictions for the 1680 small molecules profiled in LINCS in at least four cell lines consisted of 3333 unique human genes. We used a python script (available on github[[6]](#footnote-6)) to mine the PDB for structures of these genes via its RESTful Web Service interface[[7]](#footnote-7) using Uniprot primary gene name as the search criteria. Crystal structures were available for 1245 of the 3333 human genes in our analysis. The mean and median numbers of structures for these 1245 genes were 11 and 3, respectively. We then analyzed the structures for each gene and selected representative structures that would be used for docking. Representative structure selection was performed automatically using a procedure (explained below) that attempts to optimize sequence coverage, structural resolution, and structural diversity.

To select representative structures, we first divided each gene’s structures into “high” and “low” resolution categories using a 2.0 Å threshold. Small structures with less than 20 amino acids were discarded. We then used a greedy algorithm to assess sequence coverage for the remaining structures and select (as representative) the fewest and highest resolution structures that would cover the most of the protein sequence. Redundant structures, defined as structures that did not contain at least 10 residues that were not contained in any of the larger or higher resolution structures, were discarded unless they represented a unique conformation of the protein. Protein conformation was evaluated using ProDy [9] and was considered “unique” if the redundant structure had an all atom RMSD to each of the other representative structures that was above a cutoff threshold that could range between 4.0 Å and 10.0 Å. The specific value of the threshold used for each gene was chosen to try to minimize the number of redundant structures that would be docked against, and higher cutoffs were used for genes that had many redundant structures representing different conformations. After selection, the mean and median numbers of representative structures per gene were 2 and 1, respectively. Each representative structure consisted of exactly one amino acid chain and coordinated ions but without cocrystal ligands or crystallographic waters. We note that this automated procedure is not necessarily tailored to produce representative structures for functional oligomers, since only one chain is considered at a time.

**Docking procedure**

Compounds were docked to representative structures of their predicted targets with smina [10], using default exhaustiveness and a 6 Å buffer to define the box around each potential binding site. Docked poses across predicted binding sites [11] on a given target were compared and the highest scoring pose of each compound was selected for further analyses [10, 12-14] and comparison to other targets/compounds.

**Comparison to previous expression perturbation target prediction methods**

Unlike our method which uses both drug-induced and knockdown-induced mRNA expression perturbations, previous target prediction methods analyzed only the drug data within the context of protein interaction networks [15, 16]. As their primary measurement of prediction accuracy, these works generally report the aggregate Area Under the Curve (AUC) of their gene rankings across all validation compounds. The studies mentioned above achieve AUC values of 0.9 and higher in ranking between 11,000 and 18,000 potential gene targets for each compound. To compare these results against our method, we examined the reported AUC curves and calculated the percentage of compounds for which the correct target was ranked within the top 100 potential targets. Both studies achieved top-100 accuracy of 20-21%.

**Experimental Assays involving HRAS and KRAS**

*Surface Plasmon Resonance Spectroscopy:* SPR binding experiments were performed on a Biacore S200 instrument (GE, Piscatawy, NJ). Neutravidin (Pierce) was coupled to the carboxymethylated dextran surface of a CM5 sensor chip (GE, Piscatawy, NJ) using standard amine coupling chemistry to capture approximately 10,000 RU. Avi-tagged HRAS and KRAS GDP were captured on flows 2, 3 4 with densities of 2450, 2550 and 2960 RU respectively. A titration series of compounds 6, 7, and 12 diluted from 200 – 0.78 µM (seven 2-fold serial dilutions) and compound 15 diluted from 100 – 1.56 µM (six 2-fold serial dilutions) were prepared in 20mM Hepes, 150mM NaCl, 5mM MgCl2, 1mM TCEP, 0.01% Tween 20, 5% DMSO, 5µM GDP, pH 7.4. A positive control for KRAS-GDP of 250 µM DCAI was included. All compounds were injected over all flow cells at 30 µl/min. The data was processed by subtracting binding responses on the reference flow cells, buffer injections and in addition samples were also corrected for DMSO mismatches using a DMSO standard curve.

*Protein production:* Avi-HRAS(1-189) and Avi-KRAS4b(2-188) were expressed in *E. coli* as His6-MBP-tev-Avi-HRAS(1-189) and His6-MBP-tev- Avi-KRAS4b(2-188), respectively, and purified essentially as previously described [17] for a His6-MBP-tev-fusion protein.

**Experimental assays involving CHIP**

*Materials:* Rabbit anti-GST polyclonal antibody conjugated to HRP was purchased from Abcam (ab3416), mouse anti-ubiquitin monoclonal antibody was purchased from Santa Cruz Biotechnology (sc-8017), and horse anti-mouse polyclonal antibody conjugated to HRP was purchased from Cell Signaling Technology (7076S). E2 enzyme UbcH5b and recombinant human ubiquitin, and methylated human ubiquitin were obtained from Boston Biochem (E2-662, U-100H, and U-501, respectively).

*Protein purifications*: His-Ube1, His-CHIP, GST-Hsc70395-646, and GST-AT-3 JD were expressed in and purified from *E. coli* BL21(DE3) competent cells (New England Biolabs). Ube1/PET21d was a gift from Dr. Cynthia Wolberger (Addgene plasmid #34965) [18], pET151/D-TOPO CHIP and pGST‖2 Hsc70395-646 were gifts from Dr. Saurav Misra [19, 20], and pGEX6p1 AT-3 JD was a gift from Dr. Matthew Scaglione [21, 22]. Transformed cultures were incubated in Luria broth with 100 µg/mL ampicillin at 37°C and shaken at 225 rpm until an OD600 of 0.3 was attained. Protein expression was then induced with 500µM isopropyl β-D-1-thiogalactopyranoside (IPTG) and cultures were incubated for 24 hrs. at 18°C (15°C for cells expressing GST-Hsc70395-646 or GST-AT-3 JD) before the cells were harvested at 5000 rpm for 10 min at 4°C using an F7S-4x1000y rotor for the Sorvall RC-5B Plus Superspeed centrifuge. Cell pellets were stored at -80°C.

Cells harboring His-Ube1 or His-CHIP were thawed and lysed by incubation in lysis buffer (10 mM imidazole, 50 mM NaPO4 pH 8, 300 mM NaCl, 5 mM 2-mercaptoethanol, 0.25% Triton-100X, 2 mg/mL lysozyme) for 30 min on ice followed by sonication. Purification of Ube1 required addition of protease inhibitors (1% PMSF, 0.2% leupeptin, 0.1% pepstatin A) during lysis and throughout purification. After centrifugation, lysates were applied to Ni-NTA agarose resin (Qiagen), the column was washed with 30 mM imidazole, and proteins were eluted with 200 mM imidazole. Peak fractions containing His-Ube1 were pooled, dialyzed into 20 mM HEPES pH 7.4, 20 mM NaCl, and further purified by anion exchange chromatography over DEAE-Sepharose (GE Healthcare). Bound protein was eluted with a 50-300 mM NaCl gradient. Purified His-Ube1 and His-CHIP were dialyzed into 50 mM HEPES pH 7, 50 mM NaCl, and His-CHIP was further concentrated by centrifugal filtration (Millipore).

Cells harboring GST-Hsc70395-646 or GST-AT-3 JD were similarly thawed and lysed by incubation in lysis buffer (50 mM Tris pH 7.5, 150 mM NaCl, 5 mM 2-mercaptoethanol, 0.25% Triton-100X, 2 mg/mL lysozyme, with protease inhibitors) followed by sonication. After centrifugation, lysates were applied to glutathione agarose (Sigma), the column was washed, and proteins were eluted in 6.8 mg/mL reduced glutathione. Peak fractions for each substrate were pooled and dialyzed into 50 mM HEPES pH 7, 50 mM NaCl.

After isolation, the purity of all proteins was verified by SDS-PAGE followed by Coomassie Brilliant Blue staining. Protein concentration was determined by either Bradford (Bio-Rad) or BCA (Thermo Scientific) protein concentration assays. Purified proteins were flash frozen in liquid nitrogen and stored at -80°C.

*Fluorescence polarization assay:*Fluorescence polarization (FP) studies were carried out as previously described [23]. Briefly, the FP tracer was composed of a peptide derived from Hsp72/HSPA1A (GSGPTIEEVD) that was coupled at the N-terminus to 5-carboxyfluorescein (5-FAM) via an aminohexanoic acid spacer. This tracer (KD ~ 0.51 ± 0.03 µM) was used in a competition FP format to estimate binding to CHIP. Tracer concentration was 1 µM, and the CHIP concentration was 0.5 µM in a total volume of 20 µL in 50 mM HEPES, 10 mM NaCl, 0.01% Triton X-100, pH 7.4. The final DMSO concentration was approximately 1%. After mixing the components, each black 384 well plate (Corning) was covered from light and incubated at room temperature for 30 min. Polarization values were measured at Excitation 485 nm and Emission 530 nm using a Molecular Devices Spectramax M5 plate reader (Sunnyvale, CA). Data were analyzed using GraphPad Prism 6 software.

*CHIP in vitro ubiquitination assay:*Reactions were initiated by pre-incubating 125 nM Ube1, 1 µM UbcH5b, and 200 µM ubiquitin for 30 min at 37°C in 50 mM HEPES pH 7.0, 50 mM NaCl, 2 mM ATP, and 4 mM MgCl2. In a separate reaction tube, 10 µM purified CHIP and up to 500 µM compound dissolved in DMSO were combined and incubated for 15 min on ice, followed by the addition of 3 µM of either GST-Hsc70395-646 or GST-AT-3 JD, which served as substrates for CHIP-dependent ubiquitination. DMSO in these reactions was <5%. After pre-incubation, the ubiquitin-charged E1/E2 mixture was dispensed after which all reactions proceeded for 15 min at 37°C. Reactions were quenched by addition of SDS sample buffer supplemented with 50 mM EDTA, 20 mM DTT. Quenched reactions were resolved by 10% SDS-PAGE, transferred to nitrocellulose membranes and western blotted with either anti-GST HRP-conjugated antibody to visualize substrate ubiquitination, or anti-ubiquitin primary antibody, followed by an HRP-conjugated secondary antibody to visualize the amount of total ubiquitination. Products were visualized using a Bio-Rad ChemiDoc XRS+ imaging system and quantified using ImageJ software.

**Experimental assays involving PDK1**

*Materials:*Soluble biotin-phosphatidylinositol3,4,5-triphosphate, biotin-PIP3, labeled with biotin at sn1-position, was from Echelon Biosciences Inc. Bio-GST, used as a control in the alphascreen system, corresponds to biotinylated GST, (Perkin-Elmer). The peptide substrate T308tide (KTFCGTPEYLAPEVRR; > 75% purity) were synthesized using Pepscan.

*PDK1 constructs:* PDK1 CD (1-359) and PDK1 PH (360-556) were cloned in pEBG2T vector in frame with GST, expressed in HEK293 by transient transfection and purified using glutathione-sepharose, as described previously for different GST-fusion constructs [24].

*Alphascreen interaction assay:*The interaction between GST-PDK1 PH (10 nM) and biotin-PIP3 (20 nM) was measured using alphascreen technology (Perkin-Elmer), a bead-based proximity assay. The displacement of the interaction by Wortmannin was performed as previously described for the catalytic domain of PDK1 [25, 26]. Briefly, the assays were performed in a final volume of 25 µL in white 384-well microtiter plates (Greiner Bio-One), including the interacting partners in a buffer containing 50 mM Tris-HCl pH 7.4, 100 mM NaCl, 2 mM DTT, 0.01% (v/v) Tween-20, 0.1% (w/v) BSA, and the corresponding concentration of the compound (1% final DMSO concentration). 5 µL of beads (anti-GST conjugated acceptor beads and streptavidin-coated donor beads) at a 20 µg/ml (microg/ml) were then added to the mixture and after an incubation of 60 minutes, alphascreen counts were measured in an EnVision Multiplate reader. To set-up the assays, cross-titration experiments were performed, where the concentration of both interacting partners were varied. The concentration of binding partners in the assays were chosen so that both inhibitors and enhancers of the interaction could be identified. Controls using Bio-GST were performed to rule out unspecific effects on the biotin-GST alphascreen interaction assay system.

*PDK1 protein kinase activity assay:*The in vitro activity of PDK1 was tested using 100-300 ng purified protein, following the transfer of 32P from radiolabelled [g32P]ATP to the polypeptide substrate T308tide at room temperature (22 °C) in a mix containing 50 mM Tris pH 7.5, 0.05 mg/ml BSA, 0.1% β-mercaptoethanol, 10 mM MgCl2, 100 µM [g32P]ATP (5-50 cpm/pmol) and 0.003% Brij, as previously performed. [26]

**References**

1. Zhang M, Windheim M, Roe SM, Peggie M, Cohen P, Prodromou C, Pearl LH. Chaperoned ubiquitylation--crystal structures of the CHIP U box E3 ubiquitin ligase and a CHIP-Ubc13-Uev1a complex. Mol Cell. 2005;20(4):525-38. doi: 10.1016/j.molcel.2005.09.023. PubMed PMID: 16307917.

2. Gaulton A, Bellis LJ, Bento AP, Chambers J, Davies M, Hersey A, Light Y, McGlinchey S, Michalovich D, Al-Lazikani B, Overington JP. ChEMBL: a large-scale bioactivity database for drug discovery. Nucleic Acids Res. 2012;40(Database issue):D1100-7. doi: 10.1093/nar/gkr777. PubMed PMID: 21948594; PubMed Central PMCID: PMCPMC3245175.

3. Chatr-Aryamontri A, Breitkreutz BJ, Oughtred R, Boucher L, Heinicke S, Chen D, Stark C, Breitkreutz A, Kolas N, O'Donnell L, Reguly T, Nixon J, Ramage L, Winter A, Sellam A, Chang C, Hirschman J, Theesfeld C, Rust J, Livstone MS, Dolinski K, Tyers M. The BioGRID interaction database: 2015 update. Nucleic Acids Res. 2015;43(Database issue):D470-8. doi: 10.1093/nar/gku1204. PubMed PMID: 25428363; PubMed Central PMCID: PMCPMC4383984.

4. Keshava Prasad TS, Goel R, Kandasamy K, Keerthikumar S, Kumar S, Mathivanan S, Telikicherla D, Raju R, Shafreen B, Venugopal A, Balakrishnan L, Marimuthu A, Banerjee S, Somanathan DS, Sebastian A, Rani S, Ray S, Harrys Kishore CJ, Kanth S, Ahmed M, Kashyap MK, Mohmood R, Ramachandra YL, Krishna V, Rahiman BA, Mohan S, Ranganathan P, Ramabadran S, Chaerkady R, Pandey A. Human Protein Reference Database--2009 update. Nucleic Acids Res. 2009;37(Database issue):D767-72. doi: 10.1093/nar/gkn892. PubMed PMID: 18988627; PubMed Central PMCID: PMCPMC2686490.

5. Harris MA, Clark J, Ireland A, Lomax J, Ashburner M, Foulger R, Eilbeck K, Lewis S, Marshall B, Mungall C, Richter J, Rubin GM, Blake JA, Bult C, Dolan M, Drabkin H, Eppig JT, Hill DP, Ni L, Ringwald M, Balakrishnan R, Cherry JM, Christie KR, Costanzo MC, Dwight SS, Engel S, Fisk DG, Hirschman JE, Hong EL, Nash RS, Sethuraman A, Theesfeld CL, Botstein D, Dolinski K, Feierbach B, Berardini T, Mundodi S, Rhee SY, Apweiler R, Barrell D, Camon E, Dimmer E, Lee V, Chisholm R, Gaudet P, Kibbe W, Kishore R, Schwarz EM, Sternberg P, Gwinn M, Hannick L, Wortman J, Berriman M, Wood V, de la Cruz N, Tonellato P, Jaiswal P, Seigfried T, White R, Gene Ontology C. The Gene Ontology (GO) database and informatics resource. Nucleic Acids Res. 2004;32(Database issue):D258-61. doi: 10.1093/nar/gkh036. PubMed PMID: 14681407; PubMed Central PMCID: PMCPMC308770.

6. Navlakha S, He X, Faloutsos C, Bar-Joseph Z. Topological properties of robust biological and computational networks. J R Soc Interface. 2014;11(96):20140283. doi: 10.1098/rsif.2014.0283. PubMed PMID: 24789562; PubMed Central PMCID: PMCPMC4032542.

7. Andy Liaw MW. Classification and regression by randomforest. R news. 2002;2(3):18-22.

8. Qi Y, Bar-Joseph Z, Klein-Seetharaman J. Evaluation of different biological data and computational classification methods for use in protein interaction prediction. Proteins. 2006;63(3):490-500. doi: 10.1002/prot.20865. PubMed PMID: 16450363; PubMed Central PMCID: PMCPMC3250929.

9. Bakan A, Meireles LM, Bahar I. ProDy: protein dynamics inferred from theory and experiments. Bioinformatics. 2011;27(11):1575-7. doi: 10.1093/bioinformatics/btr168. PubMed PMID: 21471012; PubMed Central PMCID: PMCPMC3102222.

10. Koes DR, Baumgartner MP, Camacho CJ. Lessons learned in empirical scoring with smina from the CSAR 2011 benchmarking exercise. J Chem Inf Model. 2013;53(8):1893-904. doi: 10.1021/ci300604z. PubMed PMID: 23379370; PubMed Central PMCID: PMCPMC3726561.

11. Kozakov D, Grove LE, Hall DR, Bohnuud T, Mottarella SE, Luo L, Xia B, Beglov D, Vajda S. The FTMap family of web servers for determining and characterizing ligand-binding hot spots of proteins. Nat Protoc. 2015;10(5):733-55. Epub 2015/04/10. doi: 10.1038/nprot.2015.043. PubMed PMID: 25855957.

12. Ye Z, Baumgartner MP, Wingert BM, Camacho CJ. Optimal strategies for virtual screening of induced-fit and flexible target in the 2015 D3R Grand Challenge. J Comput Aided Mol Des. 2016;30(9):695-706. doi: 10.1007/s10822-016-9941-0. PubMed PMID: 27573981; PubMed Central PMCID: PMCPMC5079819.

13. Baumgartner MP, Camacho CJ. Choosing the Optimal Rigid Receptor for Docking and Scoring in the CSAR 2013/2014 Experiment. J Chem Inf Model. 2016;56(6):1004-12. doi: 10.1021/acs.jcim.5b00338. PubMed PMID: 26222931; PubMed Central PMCID: PMCPMC4744803.

14. Koes DR, Pabon NA, Deng X, Phillips MA, Camacho CJ. A Teach-Discover-Treat Application of ZincPharmer: An Online Interactive Pharmacophore Modeling and Virtual Screening Tool. PLoS One. 2015;10(8):e0134697. doi: 10.1371/journal.pone.0134697. PubMed PMID: 26258606; PubMed Central PMCID: PMCPMC4530941.

15. Isik Z, Baldow C, Cannistraci CV, Schroeder M. Drug target prioritization by perturbed gene expression and network information. Sci Rep. 2015;5:17417. doi: 10.1038/srep17417. PubMed PMID: 26615774; PubMed Central PMCID: PMCPMC4663505.

16. Laenen G, Thorrez L, Bornigen D, Moreau Y. Finding the targets of a drug by integration of gene expression data with a protein interaction network. Mol Biosyst. 2013;9(7):1676-85. doi: 10.1039/c3mb25438k. PubMed PMID: 23443074.

17. Dharmaiah S, Bindu L, Tran TH, Gillette WK, Frank PH, Ghirlando R, Nissley DV, Esposito D, McCormick F, Stephen AG, Simanshu DK. Structural basis of recognition of farnesylated and methylated KRAS4b by PDEdelta. Proc Natl Acad Sci U S A. 2016;113(44):E6766-E75. doi: 10.1073/pnas.1615316113. PubMed PMID: 27791178; PubMed Central PMCID: PMCPMC5098621.

18. Berndsen CE, Wolberger C. A spectrophotometric assay for conjugation of ubiquitin and ubiquitin-like proteins. Anal Biochem. 2011;418(1):102-10. doi: 10.1016/j.ab.2011.06.034. PubMed PMID: 21771579; PubMed Central PMCID: PMCPMC3178097.

19. Zhang H, Amick J, Chakravarti R, Santarriaga S, Schlanger S, McGlone C, Dare M, Nix JC, Scaglione KM, Stuehr DJ, Misra S, Page RC. A bipartite interaction between Hsp70 and CHIP regulates ubiquitination of chaperoned client proteins. Structure. 2015;23(3):472-82. doi: 10.1016/j.str.2015.01.003. PubMed PMID: 25684577; PubMed Central PMCID: PMCPMC4351142.

20. Sheffield P, Garrard S, Derewenda Z. Overcoming expression and purification problems of RhoGDI using a family of "parallel" expression vectors. Protein Expr Purif. 1999;15(1):34-9. doi: 10.1006/prep.1998.1003. PubMed PMID: 10024467.

21. Todi SV, Scaglione KM, Blount JR, Basrur V, Conlon KP, Pastore A, Elenitoba-Johnson K, Paulson HL. Activity and cellular functions of the deubiquitinating enzyme and polyglutamine disease protein ataxin-3 are regulated by ubiquitination at lysine 117. J Biol Chem. 2010;285(50):39303-13. doi: 10.1074/jbc.M110.181610. PubMed PMID: 20943656; PubMed Central PMCID: PMCPMC2998082.

22. Faggiano S, Menon RP, Kelly GP, McCormick J, Todi SV, Scaglione KM, Paulson HL, Pastore A. Enzymatic production of mono-ubiquitinated proteins for structural studies: The example of the Josephin domain of ataxin-3. FEBS Open Bio. 2013;3:453-8. doi: 10.1016/j.fob.2013.10.005. PubMed PMID: 24251111; PubMed Central PMCID: PMCPMC3829987.

23. Assimon VA, Southworth DR, Gestwicki JE. Specific Binding of Tetratricopeptide Repeat Proteins to Heat Shock Protein 70 (Hsp70) and Heat Shock Protein 90 (Hsp90) Is Regulated by Affinity and Phosphorylation. Biochemistry. 2015;54(48):7120-31. doi: 10.1021/acs.biochem.5b00801. PubMed PMID: 26565746.

24. Dettori R, Sonzogni S, Meyer L, Lopez-Garcia LA, Morrice NA, Zeuzem S, Engel M, Piiper A, Neimanis S, Frodin M, Biondi RM. Regulation of the interaction between protein kinase C-related protein kinase 2 (PRK2) and its upstream kinase, 3-phosphoinositide-dependent protein kinase 1 (PDK1). J Biol Chem. 2009;284(44):30318-27. doi: 10.1074/jbc.M109.051151. PubMed PMID: 19723632; PubMed Central PMCID: PMCPMC2781587.

25. Zhang H, Neimanis S, Lopez-Garcia LA, Arencibia JM, Amon S, Stroba A, Zeuzem S, Proschak E, Stark H, Bauer AF, Busschots K, Jorgensen TJ, Engel M, Schulze JO, Biondi RM. Molecular mechanism of regulation of the atypical protein kinase C by N-terminal domains and an allosteric small compound. Chem Biol. 2014;21(6):754-65. doi: 10.1016/j.chembiol.2014.04.007. PubMed PMID: 24836908.

26. Schulze JO, Saladino G, Busschots K, Neimanis S, Suss E, Odadzic D, Zeuzem S, Hindie V, Herbrand AK, Lisa MN, Alzari PM, Gervasio FL, Biondi RM. Bidirectional Allosteric Communication between the ATP-Binding Site and the Regulatory PIF Pocket in PDK1 Protein Kinase. Cell Chem Biol. 2016;23(10):1193-205. doi: 10.1016/j.chembiol.2016.06.017. PubMed PMID: 27693059.

1. http://support.lincscloud.org/hc/en-us/sections/200437157-L1000-Assay [↑](#footnote-ref-1)
2. http://code.lincscloud.org/ [↑](#footnote-ref-2)
3. https://www.ebi.ac.uk/chembl/ [↑](#footnote-ref-3)
4. https://github.com/cmap/l1ktools [↑](#footnote-ref-4)
5. http://geneontology.org/page/go-enrichment-analysis [↑](#footnote-ref-5)
6. https://github.com/npabon/generate_gene_models [↑](#footnote-ref-6)
7. https://www.rcsb.org/pdb/software/rest.do [↑](#footnote-ref-7)
